# Tolerogenic Immunomodulation by PEGylated Antigenic Peptides

**DOI:** 10.3389/fimmu.2020.529035

**Published:** 2020-10-09

**Authors:** Jennifer Pfeil, Mario Simonetti, Uta Lauer, Rudolf Volkmer, Bianca von Thülen, Pawel Durek, Ralf Krähmer, Frank Leenders, Alf Hamann, Ute Hoffmann

**Affiliations:** ^1^ Experimental Rheumatology, Deutsches Rheuma-Forschungszentrum, a Leibniz Institute (DRFZ), Berlin, Germany; ^2^ Department of Rheumatology and Clinical Immunology, Charité Universitätsmedizin, Berlin, Germany; ^3^ Institute for Medical Immunology, Charité Universitätsmedizin, Berlin, Germany; ^4^ Celares GmbH, Berlin, Germany

**Keywords:** immune tolerance, autoimmunity, peptide vaccination, nanoparticles, regulatory T cells

## Abstract

Current treatments for autoimmune disorders rely on non-specific immunomodulatory and global immunosuppressive drugs, which show a variable degree of efficiency and are often accompanied by side effects. In contrast, strategies aiming at inducing antigen-specific tolerance promise an exclusive specificity of the immunomodulation. However, although successful in experimental models, peptide-based tolerogenic “inverse” vaccines have largely failed to show efficacy in clinical trials. Recent studies showed that repetitive T cell epitopes, coupling of peptides to autologous cells, or peptides coupled to nanoparticles can improve the tolerogenic efficacy of peptides, suggesting that size and biophysical properties of antigen constructs affect the induction of tolerance. As these materials bear hurdles with respect to preparation or regulatory aspects, we wondered whether conjugation of peptides to the well-established and clinically proven synthetic material polyethylene glycol (PEG) might also work. We here coupled the T cell epitope OVA^323–339^ to polyethylene glycols of different size and structure and tested the impact of these nano-sized constructs on regulatory (Treg) and effector T cells in the DO11.10 adoptive transfer mouse model. Systemic vaccination with PEGylated peptides resulted in highly increased frequencies of Foxp3^+^ Tregs and reduced frequencies of antigen-specific T cells producing pro-inflammatory TNF compared to vaccination with the native peptide. PEGylation was found to extend the bioavailability of the model peptide. Both tolerogenicity and bioavailability were dependent on PEG size and structure. In conclusion, PEGylation of antigenic peptides is an effective and feasible strategy to improve Treg-inducing, peptide-based vaccines with potential use for the treatment of autoimmune diseases, allergies, and transplant rejection.

## Introduction

The limited efficacy and increased risk of opportunistic infection associated with classical immunosuppressants or modern biologics still represent a challenge in the therapy of immune-mediated diseases such as autoimmunity, allergy, and transplant rejection. The resulting medical need has led to a revival of the old idea of antigen-specific therapies under the headline of “inverse vaccination” (1, 2).

Numerous studies have demonstrated that the administration of soluble antigens or immunodominant peptides under non-inflammatory conditions can indeed induce a state of unresponsiveness. Clonal deletion, induction of anergy, or modification in the cytokine profile of pro-inflammatory effector cells and, notably, induction and expansion of regulatory T cells (Tregs) were discussed as possible mechanisms in induced tolerance (3–8). Tregs play a pivotal role in maintaining self-tolerance and in modulating immune responses in inflammatory diseases. Foxp3^+^ Tregs are generated either as a distinct lineage in the thymus during T cell development (tTregs) or in the periphery by conversion of conventional naive Foxp3^-^ T cells (pTregs) upon antigen exposure (9).

For use in tolerogenic vaccination, native protein antigens were first used and provide a broad spectrum of potential antigenic sites, while peptides representing immunodominant epitopes (as far as identified) are more “druggable” with regard to costs and regulatory issues. However, in part of animal studies and especially in first clinical trials in the human, peptide vaccination has not met the expectations. Both lack of efficacy and adverse effects, resulting in exacerbation of the autoimmune disease, were reported (10–13).

The efficacy of small peptides is restricted by their rapid excretion through the kidney or enzymatic degradation (14). This provoked further studies aiming to improve the tolerogenic potential of peptides by coupling to autologous cells (15, 16), to carrier proteins (17), coupling to or encapsulation of peptides into different kinds of nanoparticles (18–21), and others as reviewed in (7, 22–25).

In a preceding study, a tolerogenic effect of peptide epitopes was only observed when the peptide was multimerized or coupled to a repetitive carrier protein, but not as native peptide (17). This let us hypothesize that just molecular size is an important parameter in the tolerogenic efficacy. We previously reported that coupling of the OVA-peptide to dendritic oligoglycerol (OG)- or polyglycerol (PG)- nanoparticles resulted for some conjugates in an efficient induction of antigen-specific Tregs *in vivo* (20). In this study, linker chemistry, size, and structure were found to play a critical role for the tolerogenic potential of the conjugates.

Coupling of therapeutic proteins to another synthetic polymer, polyethylene glycol (PEG) is already widely applied for the optimization of biologics (26–28). PEG is a synthetic polymer composed of repetitive ethylene oxide subunits, either in linear form or as branched polymers. The covalent attachment of PEG (PEGylation) improves the pharmacokinetic and pharmacodynamic profile of biomolecules by increasing serum half-life. As PEG entangles around the peptide/protein surface and forms hydrogen bonds with the encircling water molecules, additional effects such as increased solubility of the conjugates, improved resistance to proteolysis, or reduced immunogenicity of biomolecules can be observed (29–32). We therefore analyzed here whether coupling of antigenic peptides to nano-sized PEG would improve efficacy and safety of vaccination. Numerous PEGylated biomolecules have already been approved by the Food Drug Administration (FDA) and the European Medicines Agency (EMA) for human use as ingredients of foods, cosmetics, and pharmaceuticals including topical, parenteral, and nasal formulations.

We therefore reasoned that coupling of peptides to PEG could provide not only favorable properties, notably increased size, and hence prolonged bioavailability but also safety and stability. Most important, coupling to PEG would build upon a clinically proven and well controllable chemistry. We used the transgenic DO11.10 adoptive transfer model (33) to test efficacy and safety of vaccination with PEG-coupled OVA-peptide. While *in vitro*, conjugates were much less efficient in activation of specific T cells than the native peptide, the contrary was true *in vivo*, correlating with an increased bioavailability of the conjugate. Systemic vaccination with PEGylated peptide resulted in highly increased frequencies of Foxp3^+^ Tregs as well as reduced frequencies of pro-inflammatory (TNF^+^) antigen-specific T cells, compared to vaccination with the native peptide. These data suggest that coupling of peptide to PEG might result in an improved tolerogenic activity.

## Material and Methods

### Peptides

OVA-peptide 323–339 (pOVA: ISQAVHAAHAEINEAGR), cysteine-modified pOVA (CG-ISQAVHAAHAEINEAGR) and cysteine-modified MOG-peptide 35–55 (CβA-MEVGWYRSPFSRVVHLYRNGK) were synthesized in house. N-terminally cysteine-modified peptides were used for conjugation to maleimido-functionalized PEG polymers.

### Preparation of PEGylated Peptides

(For formulas see **Supplementary Figure 1**).

#### Synthesis of pOVA-PEG

A 12-kDa PEG-maleimide was obtained from Quanta Biodesign (USA), 20 and 40 kDa PEG-maleimide from NOF Corporation (Japan). To couple PEG to the CG-modified N-terminus of pOVA_323–339_, 450 mg of 20 kDa PEG-maleimide or 900 mg of 40 kDa PEG-maleimide and 40 mg CG-pOVA_323–339_ were reacted in 40 mL of a 50 mmol/L of sodium phosphate buffer (pH 7.0). The reaction mixture was stirred at RT for 1 h. Progress of the reaction was monitored by RP-HPLC.

#### Synthesis of pOVA-PEG-Tetramer

To prepare a pOVA-PEG-tetramer, 54 mg of CG-pOVA_323–339_ were reacted with 7 mg of 4arm-PEG-maleimide (celares GmbH, Germany) in 4.5 ml of 50 mmol/L of sodium phosphate buffer (pH 7.2). The reaction mixture was stirred at RT for 2 h. Progress of the reaction was monitored by RP-HPLC.

#### Synthesis of pMOG-PEG20

To couple PEG to the C-modified N-terminus of pMOG, 157 mg of 20 kDa PEG-maleimide (NOF Corporation, Japan) and 20 mg pMOG were reacted in 20 mL of a 20 mmol/L of sodium phosphate buffer pH 7.2 containing 250 µmol/L of Tris(2-carboxyethyl)phosphine. The reaction mixture was stirred at RT for 1 h. Progress of the reaction was monitored by RP-HPLC.

#### Purification of Peptide-PEG-Conjugates

Crude peptide-PEG-conjugates were purified by cation-exchange chromatography using MacroCap SP on an Äkta chromatography system. pOVA-PEG-conjugates were bound to the resin in 20 mmol/L of sodium citrate buffer (pH 3.0) as eluent and pMOG-PEG20 in 20 mmol/L of sodium acetate (pH 4.5), respectively. Conjugates eluted with a linear sodium chloride gradient from 0 to 500 mM in 10 column volumes in the same eluent. Conjugate elution was monitored at 213 nm. Fractions containing the peptide-PEG-conjugates were pooled and desalted by dialysis against deionized water. Subsequently, conjugates were concentrated by freeze-drying. Prior to use, conjugates were reconstituted in WFI and filtered through a sterile 0.2µm filter.

#### Analysis of Peptide-PEG-Conjugates

Analysis of peptide-PEG-conjugates was performed by MALDI-ToF-MS, UV-spectrometry, and reversed phase-high performance liquid chromatography (RP-HPLC). RP-HPLC was performed on a Waters Alliance System. For the analysis of pOVA containing conjugates, an XBridge™BEH300 C18 5 µm 4.6 x 250 mm was used. Samples were applied in water containing 0.1% TFA and eluted with a linear gradient from 37.5 to 50% acetonitrile in water each with 0.1% TFA in 15 min. For the analysis of pMOG-PEG20, a Butyl C4 column 4.6 x 250 mm (5 µm) was used. Sample was eluted with a linear gradient from 70:15:15 to 50:25:25 water:acetonitrile:isopropanol each with 0.1% TFA in 10 min. PEG or peptide-PEG-conjugates were detected using an evaporating light scattering detector (ELSD). All peptide-PEG-conjugates were free of any residual unmodified peptide.

### Mice

DO11.10 OVA-TCR^+^ transgenic mice, extensively backcrossed (>10 generations) onto BALB/c background, OTIIxB6.PL and MHCII^-^/^-^ mice, extensively backcrossed (>10 generations) onto C57BL/6 background, were all housed in DRFZ breeding facility. Female BALB/c mice were purchased from Charles River and used at 9 to 13 weeks of age. Mice were maintained under specific pathogen-free conditions according to national and institutional guidelines. All experiments were approved by Landesamt für Gesundheit und Soziales (LAGeSo).

### Cell Preparation

To isolate OVA-TCR^+^ CD4^+^ cells, single cell suspensions were prepared from peripheral lymph nodes (cervical, brachial, axillary and inguinal; pLN), mesenteric lymph nodes (mLN) and spleen isolated from DO11.10 or OTIIxB6PL mice. Red blood cells were lysed in lysis buffer (0.01 M potassium bicarbonate, 0.155 M ammonium chloride, and 0.1 mM ethylenediaminetetraacetic acid (EDTA) and washed with phosphate buffered saline (PBS) supplemented with 0.2% bovine serum albumin (BSA). CD4^+^ T cells from DO11.10 mice were enriched using anti-CD4 Microbeads (RM4-5, Miltenyi Biotec, Bergisch Gladbach, Germany) according to the manufacturer’s instructions and sorted using an AutoMACS Pro (Miltenyi Biotec).

For preparation of OVA-TCR^+^ CD4^+^ MHCII^-^ cells from OTIIxBL6 mice anti-CD4-FITC and anti-MHCII-APC antibodies were added and incubated for 15 min at 4°C. Subsequently, CD4^+^ MHCII^-^ cells were enriched using anti-FITC MultisortBeads (Miltenyi Biotec) and anti-APC Microbeads (Miltenyi Biotec) according to manufacturer’s instructions. Purity of the CD4^+^ MHCII^-^ fraction was above 97%, which was verified by flow cytometric analysis.

### Antibodies and Flow Cytometry

The following antibodies were obtained from eBioscience (San Diego, CA, USA): eFluor 450-conjugated anti-CD4 (RM4-5), PeCy7-conjugated anti-CD11b (M1/70), PECy7-conjugated anti-IFN-γ (XMG1.2), PerCP-eFluor 710-conjugated anti-TNFα (MP6-XT22), eFluor 450-conjugated anti-Foxp3 (FJK-16s), and appropriate isotype controls. V500-conjugated anti-CD4 (RM4-5) antibody and PE-conjugated anti-Thy1.1 (OX-7) antibody were purchased from BD Bioscience (Heidelberg, Germany). PerCP-conjugated anti-CD11c (N418) and Pacific-Blue-conjugated anti-CD19 (6D5) antibodies were obtained from Biolegend (San Diego, USA). Cy5-conjugated anti-OVA-TCR (KJ1.26) and anti-Fcγ-receptor (2.4G2) antibodies were produced in house (DRFZ). Total rat IgG was purchased from Dianova (Hamburg, Germany). The APC-conjugated anti-CD154 antibody was obtained from Miltenyi Biotec.

To stain surface molecules cells were incubated with anti-Fcγ-receptor antibody (20 µg/ml). Cell fixation, permeabilization, and intracellular Foxp3 staining were performed using the eFluor450 anti-mouse Foxp3 (eBioscience) staining set according to manufacturer’s instructions.

For cytokine staining, cells were stimulated in cRPMI supplemented with phorbol-12-myristate 13-acetate (PMA; 10 ng/ml) and ionomycin (500 ng/ml) for 4 h at 37°C in a humidified 5% CO_2_ atmosphere. After 2 h incubation, Brefeldin A (10 µg/ml) was added. Cells were fixed by incubation with 2% paraformaldehyde (PFA) at RT. Intracellular staining was performed by 5 min pre-incubation with rat IgG in 0.5% saponin (all reagents from Sigma-Aldrich). Monoclonal rat anti-mouse cytokine antibodies were added and incubated for another 30 min in 0.5% saponin at RT. Flow cytometric analysis was performed using a FACS Canto II (BD Bioscience, Heidelberg, Germany) and Flow Jo software (Tree star, OR, USA).

### Determination of Cell Proliferation In Vitro and In Vivo

For *in vitro* proliferation assays, CD4^+^ cells from DO11.10 mice were labeled with 1 µM CFSE (5`carboxyfluorescein succinimidyl ester). The CD4 negative fraction was depleted from the remaining T cells using anti-CD90 Microbeads (Miltenyi Biotec) and AutoMACS separation according to the manufacturer’s instructions and used as antigen-presenting cells (APCs). APCs were irradiated (30 Gray) and cultured with CFSE-labeled CD4^+^ cells in a final concentration of 2x10^6^ cells/ml cRPMI (RPMI 1640 Glutamax medium, Gibco, supplemented with 10% FCS, penicillin 100 U/ml, streptomycin 100 µg/ml, 2-mercaptoethanol 1 mM, sodium pyruvate 1 mM and Hepes buffer 25 mM) 3:1 in 96-well round-bottom plates. Unconjugated pOVA or PEGylated peptides were added in concentrations as indicated. Cells were incubated for 4 days at 37°C in a humidified 5% CO_2_ atmosphere.

For determination of cell proliferation, cells were analyzed by gating on OVA-TCR^+^ CD4^+^ cells and calculating the geometrical mean of the fluorescence intensity (GMFI) of the CFSE signal. Fold CFSE dilution was determined as GMFI (PBS control)/GMFI (sample) and plotted on a log_2_-scale where every log step represents one division step.

### Th1 Polarization

CD4^+^ T cells were isolated from DO11.10 mice *via* MACS-sorting as above and co-cultured 1:3 with APCs (CD4^-^ CD90^-^) (final concentration: 2 x 10^6^ cells/ml). Cells were incubated with 1 µg/ml of pOVA, IFN-γ (20 ng/ml) (R&D Systems, McKinley, USA), IL12 (5 ng/ml) (R&D), and anti-IL4-antibody (11B11, produced in house) (5 µg/ml). After 6 days, cells were harvested, and IFN-γ expression was assessed *via* flow cytometry.

### Adoptive T Cell Transfer Assays

#### Adoptive Transfer of Ex Vivo Isolated OVA-TCR^+^ CD4^+^ Cells

On day 0, 5 × 10^6^ CFSE-labeled CD4^+^ cells from DO11.10 were adoptively transferred intravenously (*i.v.*) into syngeneic BALB/c mice. After 24 hours, mice were treated by a single i.v. injection of PBS (control), unconjugated pOVA or equimolar amounts of PEG-conjugated pOVA as indicated. In some experiments, mice were also treated with unconjugated pOVA + 50 µg of lipopolysaccharide (LPS) or pOVA-PEG + 50 µg of LPS. On day 7, splenocytes were isolated, and OVA-TCR^+^ CD4^+^ T cells were identified by an anti-KJ1.26 antibody, recognizing the OVA-specific TCR on transferred DO11.10 cells.

In experiments using MHC^-^/^-^ bone marrow (BM) chimeras, 5 x 10^6^ CFSE-labeled OVA-TCR^+^ CD4 MHCII^-^ cells from OTIIxB6PL mice were adoptively transferred either into wt or MHCII^-^/^-^ BM chimeras. 6 days after antigen exposure, splenocytes were isolated, and OVA-TCR^+^ CD4^+^ T cells were identified by Th1.1 (CD90) on transferred OTIIxB6PL cells.

#### Adoptive Transfer of Treg-Depleted CD4^+^ Cells

To investigate whether Tregs are expanded or induced *de novo*, purified CFSE-labeled CD4^+^ CD25^-^ CD103^-^ T cells from DO11.10 mice were adoptively transferred i.v. into BALB/c mice. After 24 h, mice were treated by a single i.v. injection of PBS (control), unconjugated pOVA at a dose of 5 µg per mouse or equimolar amounts of PEGylated pOVA. On day 7, different lymphatic organs were isolated and OVA-specific CD4^+^ T cells were identified by an anti-KJ1.26 antibody.

#### Adoptive Transfer of Antigen-Specific Th1 Cells

5 × 10^6^ purified polarized CFSE-labeled Th1 cells from DO11.10 mice were adoptively transferred i.v. into BALB/c mice. After 24 h, mice were treated by a single i.v. injection of PBS (control), unconjugated pOVA at a dose of 5 µg per mouse or equimolar amounts of PEGylated pOVA. On day 5, splenocytes were isolated, and OVA-specific Th1 cells were identified by an anti-KJ1.26 antibody.

#### Delayed-Type Hypersensitivity (DTH) Model

pOVA-specific CD4^+^ cells were adoptively transferred into BALB/c mice, which were treated i.v. with PBS (control), 5 μg of pOVA or equimolar amounts of pOVA-PEG20 24 h later (tolerization). To induce the effector phase, *in vitro* generated pOVA-specific Th1 cells were transferred into the recipients on day 7. After 24 h, pOVA in IFA or PBS/IFA were injected into the left or right hind footpad, respectively. Footpad swelling was monitored for 8 consecutive days and expressed as Δ footpad thickness.

### Analysis of Bioavailability

To test for the presence of bioactive material in the organism after an extended period of time (bioavailability), recipients were treated with pOVA or equimolar amounts of PEGylated peptides 3 days before transfer of CFSE-labeled OVA-specific CD4^+^ cells. Proliferation was assessed 6 days after CD4^+^ T cell transfer by dilution of CFSE intensity using flow cytometry.

### Generation of MHCII^-^/^-^ Chimeric Mice

Wt C57BL/6 recipient mice were lethally irradiated with 10.5 Gray. After 24 h mice were reconstituted by a single i.v. injection of 5 x 10^6^ BM cells from either MHCII^-^/^-^ mice or wt C57BL/6 BM cells (control). Therefore, BM cells from MHCII^-^/^-^ and wt C57BL/6 (control) were enriched by depletion of CD90^+^ cells using anti-CD90 Microbeads according to the manufacturer’s instructions. Starting one week prior and ending 7 weeks after BM transplantation, BM chimeras continuously received 0.4% enrofloxacin (Baytril**^®^**, Bayer, Leverkusen, Germany). After 7 weeks, reconstitution with MHCII^-^/^-^ BM cells was confirmed using flow cytometry (**Supplementary Figure 6**). Within the tissues radioresistant non-hematopoietic APCs remained MHCII^+^, whereas recipient hematopoietic APCs were replaced by MHCII^-^/^-^ donor cells (34).

### Statistics

**Figure 1:**
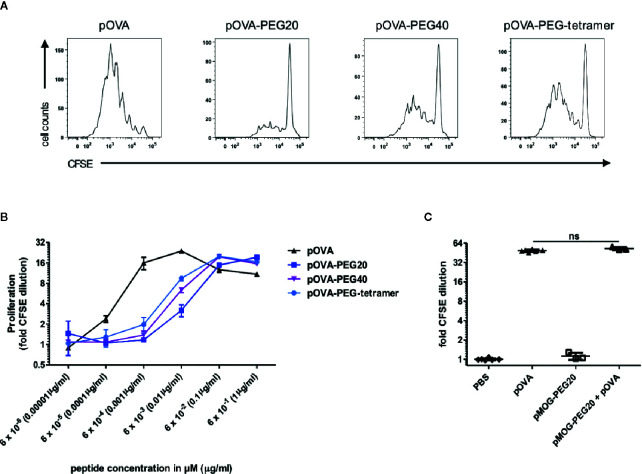
Stimulatory capacity of PEGylated peptides *in vitro.* **(A)** Representative histograms of CFSE dilution following stimulation with 6 nM pOVA or equimolar amounts (based on peptide amount) of PEGylated peptides.** (B)** Dose-response curves of peptide and conjugates. CFSE labeled OVA-specific CD4^+^ T cells from DO11.10 mice were cultured with irradiated APCs (1:3) for four days. pOVA and equimolar amounts of pOVA-PEG conjugates (based on peptide amount) were added as indicated. For analysis of proliferation, cells were gated on OVA-TCR (KJ1.26)^+^ CD4^+^ cells and the geometrical mean of the fluorescence intensity (GMFI) of CFSE was determined. Fold CFSE dilution was calculated as: GMFI (control)/GMFI (sample); log_2_-scale. One log_2_ step is equivalent to one cell division. Data are means of triplicates ± SD and representative for at least three independent experiments. **(C)** To exclude unspecific inhibitory effects of the PEG polymer, OVA-specific CD4^+^ T cells were stimulated *in vitro* with 100 nM pOVA in presence or absence of 100 nM of the irrelevant pMOG-PEG20. After 6 days, proliferation was assessed by flow cytometry. n = 3–6 from two independent experiments. Statistical testing was performed using the nonparametric Mann Whitney test and Holm-Bonferroni correction for multiple comparisons. ns, non-significant. For **(B)**, dose response curve fitting and statistical analysis is provided in **Supplementary Figure 2**.

Data was analyzed using PRISM 5, 7 or 8 (GraphPad, La Jolla, CA, USA). Significance was determined with the non-parametric Mann Whitney U test followed by Holm-Bonferroni correction. Differences were considered as statistically significant with p ≤ 0.05 (*), very significant with p ≤ 0.01 (**), and extremely significant with p ≤ 0.001 (***) or marked as non-significant (ns). Data of *in vitro* dose response (**Figure 1B**) were additionally analyzed by curve fitting using global nonlinear fitting of the default Hill model, as described in detail in **Supplementary Figure 2**. For footpad swelling curves (**Figure 11**), significance was determined on all values using the Mann Whitney U test followed by Holm-Bonferroni correction.

## Results

### Biologic Activity of PEGylated Peptides

In order to test whether T cell stimulation capacity is preserved after conjugation to different PEG carriers, the model peptide ovalbumin peptide 323–339 (pOVA) was either monovalently conjugated to linear PEG molecules [PEG20 (20 kDa) or PEG40 (40 kDa)] or polyvalently coupled to a branched PEG-tetramer (3.1 kDa). PEG-peptide conjugates were analyzed *in vitro* and *in vivo* for their capacity to activate transgenic, OVA-specific CD4^+^ T cells from DO11.10 mice in the presence of antigen-presenting cells (APCs).

#### Reduced Activation of Antigen-Specific CD4^+^ T Cells by PEGylated Peptides *In Vitro*

To determine activation of OVA-TCR^+^ CD4^+^ T cells, cells were labeled with carboxyfluorescein succinimidyl ester (CFSE) and proliferation was assessed by dilution of CFSE intensity in daughter cells on day 4 of culture. T cells were cultured in presence of APCs (CD90^-^ CD4^-^) and different concentrations of pOVA or equimolar amounts (based on peptide amount) of different pOVA-PEG conjugates (pOVA-PEG20, pOVA-PEG40, pOVA-PEG-tetramer). All conjugates were able to activate antigen-specific CD4^+^ T cells (**Figures 1A, B**). However, 21 to 91 fold higher concentrations of PEGylated peptides were required to induce half-maximal proliferation compared to unmodified pOVA (for curve fitting and statistics see **Supplementary Figure 2**).

In order to exclude unspecific effects of the macromolecular carrier PEG, PEG20 was conjugated to an irrelevant peptide (epitope 35–55 of myelin oligodendrocyte glycoprotein (pMOG)), which is not recognized by OVA-TCR^+^ CD4^+^ T cells. PEG conjugated to pMOG did not induce activation of OVA-specific T cells. Furthermore, pMOG-PEG20 did not influence pOVA-induced T cell proliferation when added to pOVA-stimulated cultures *in vitro* (**Figure 1C**). This proved that the reduced activity is due to impaired presentation or recognition of the conjugated peptide, while PEG does not have inhibitory effects per se.

#### PEGylated Peptides Efficiently Activate Antigen-Specific CD4^+^ T Cells *In Vivo*

**Figure 2:**
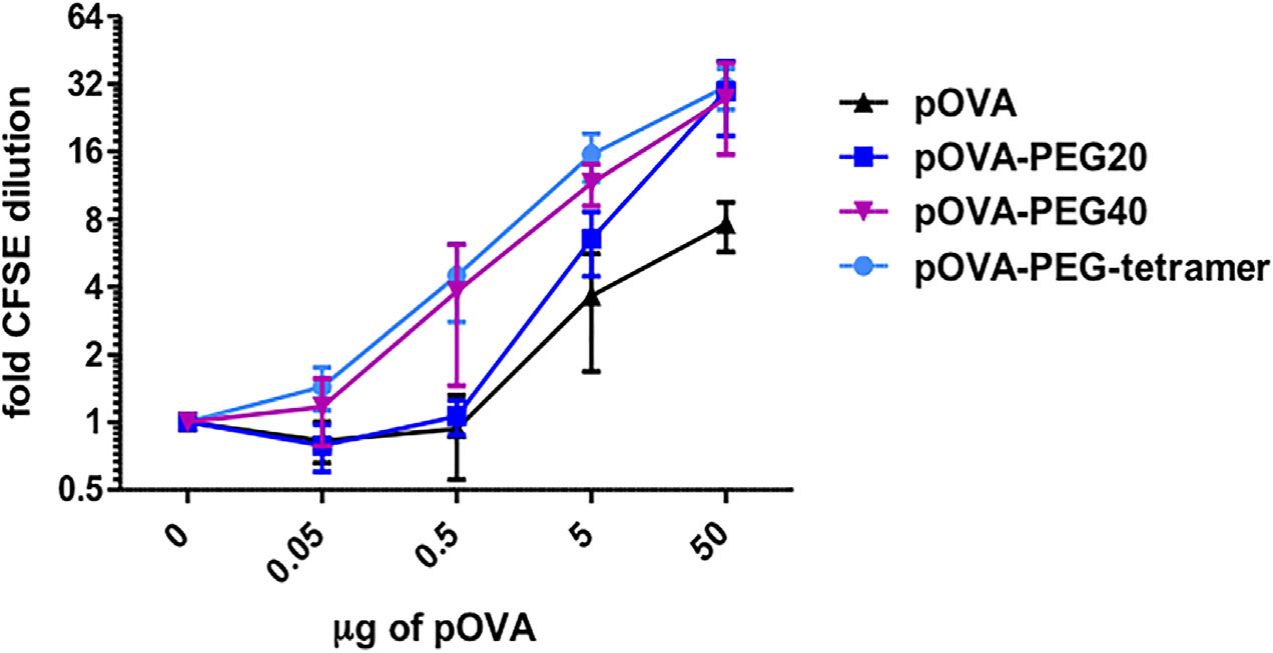
PEGylated peptides efficiently activate antigen-specific CD4^+^ T cells *in vivo*. CFSE-labeled CD4^+^ T cells from DO11.10 mice were transferred into BALB/c mice. After 24 h recipients received i.v. PBS (control), indicated amounts of pOVA or equimolar amounts (based on peptide amount) of pOVA-PEG20, pOVA-PEG40 or pOVA-PEG-tetramer. 5 µg of pOVA are equivalent to 2.8 nM (and 250 µg/kg) per mouse. After 6 days, proliferation was assessed by flow cytometry (for representative dot plots, see **Figure 4**). Displayed is the mean x-fold CFSE dilution ± SD gated on OVA-TCR^+^ CD4^+^ splenocytes from at least two independent experiments. n = 4–6; PBS, n = 10.

To investigate the effect of PEGylated peptides on activation of antigen-specific CD4^+^ T cells *in vivo*, an adoptive transfer system was used. OVA-specific CFSE-labeled CD4^+^ T cells from transgenic DO11.10 mice were transferred into syngeneic BALB/c mice. After 24 h, recipients were treated by a single i.v. injection of PBS (control), indicated amounts of pOVA or equimolar amounts (based on peptide amount) of pOVA-PEG20, pOVA-PEG40 or pOVA-PEG-tetramer. After 6 days, splenocytes of recipients were isolated and OVA-specific CD4^+^ T cells were analyzed by flow cytometry. In contrast to the limited capacity of PEGylated peptides stimulating OVA-TCR^+^ CD4**^+^** T cells *in vitro*, T cells strongly proliferated when pOVA-PEG conjugates were administered *in vivo* (**Figure 2**). pOVA-PEG20 and especially pOVA-PEG40 and pOVA-PEG-tetramer were significantly more effective in activating OVA-specific cells than unconjugated pOVA.

#### Conjugation of Peptide to PEG Prolongs Availability *In Vivo*

**Figure 3:**
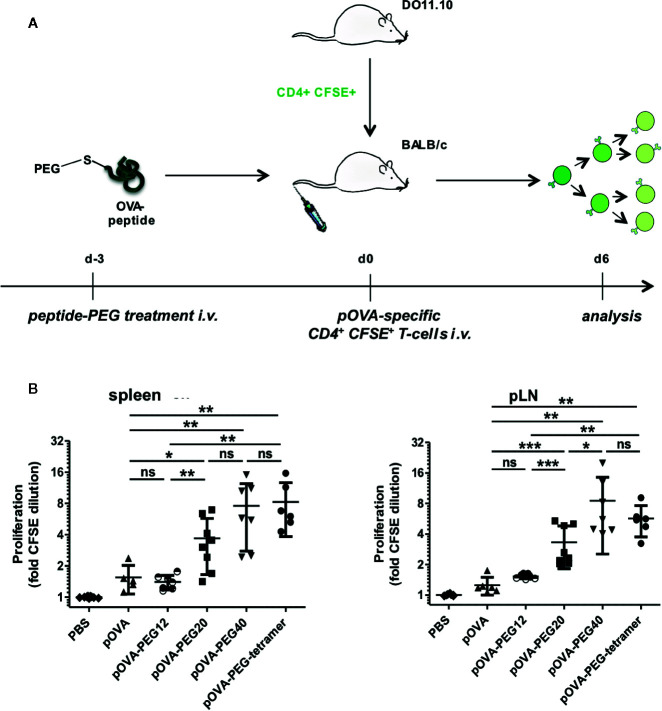
Conjugation of pOVA to the macromolecular carrier PEG extends peptide bioavailability. **(A)** Set-up for analysis of the bioavailability. **(B)** OVA-specific CD4^+^ CFSE-labeled T cells were transferred into BALB/c mice three days after i.v. peptide vaccination with unconjugated pOVA or equimolar amounts of pOVA-PEG conjugates. After 6 days proliferation was assessed by flow cytometry. Cells from spleen and pLN were gated on OVA-TCR^+^ CD4^+^ T cells. Data show mean x-fold CFSE dilution ± SD from at least two independent experiments. n = 6–9. Statistical testing was performed using the nonparametric Mann Whitney test and Holm-Bonferroni correction for multiple comparisons. ns, non-significant; (*) p < 0.05; (**) p < 0.01; (***) p < 0.001.

We next addressed the question whether PEGylation leads to an extended half-life (bioavailability) of the antigenic peptide in the organism and whether size and structure of the PEG molecule matter. For this, we tested the degree of proliferation after vaccination with a single i.v. injection of pOVA or conjugates at 3 days prior to transfer of OVA-specific T cells (**Figure 3A**). Six days after cell transfer, splenocytes were isolated and proliferation of OVA-TCR^+^ CD4^+^ T cells was analyzed according to CFSE content. Little T cell proliferation was observed upon vaccination with unconjugated pOVA or a 12 kDa PEG (pOVA-PEG12), suggesting a rapid systemic clearance within the three days (**Figure 3B**). In contrast, pOVA-PEG20, pOVA-PEG40 and pOVA-PEG-tetramer led to significant proliferation. Interestingly, pOVA-PEG-tetramer induced, despite its small size (4 x 1.9 kDa pOVA + 3.1 kDa PEG; in total 11 kDa), proliferation as strong as pOVA-PEG40.

### Tolerogenic Potential of PEGylated Peptides *In Vivo*

#### Induction of Tregs

**Figure 4:**
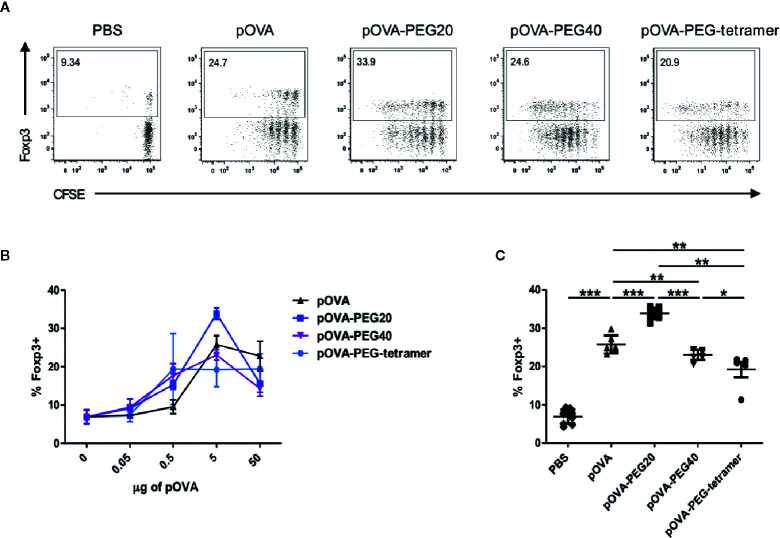
Increased frequency of regulatory Foxp3^+^ CD4^+^ T cells upon pOVA-PEG20 treatment. CFSE-labeled CD4^+^ cells from DO11.10 mice were transferred into BALB/c mice. After 24 h, mice received either PBS (control), indicated doses of pOVA or equimolar amounts of PEGylated peptides. After 6 days, Foxp3 expression was assessed using flow cytometry. Spleen cells were gated on OVA-TCR^+^ CD4^+^ T cells. **(A)** Representative FACS dot plots of Foxp3 expression following tolerization with pOVA or pOVA-PEG conjugates at a dose of 5 µg (based on peptide amount). **(B)** Dose response curve *in vivo*. Data displayed as mean ± SD of % Foxp3^+^ cells from at least two independent experiments. n = 4–8; PBS, n = 10. **(C)** Pooled data from treatment with 5 µg of pOVA or equimolar amounts of PEGylated pOVA. Mean ± SD from at least two independent experiments. Statistical testing was performed using the nonparametric Mann Whitney test and Holm-Bonferroni correction for multiple comparisons. (*) p < 0.05; (**) p < 0.01; (***) p < 0.001.

To assess the immunomodulatory effect of administration of different types of PEGylated pOVA, we analyzed the expression of Foxp3, the master transcription factor of Tregs and the cytokine profile of antigen-specific T cells upon vaccination. A significant increase in the frequency of splenic OVA-specific Foxp3^+^ regulatory T cells was observed following i.v. administration of 5 µg of (2.8 nM) native pOVA compared to PBS-treated mice (**Figures 4A–C**). The effect was antigen-specific since the frequency of Foxp3^+^ Tregs was not altered among endogenous CD4^+^ T cells lacking the transgenic TCR (data not shown). Vaccination with equimolar amounts of pOVA-PEG20, pOVA-PEG40, and pOVA-PEG-tetramer also increased Foxp3^+^ frequencies among OVA-TCR^+^ cells (**Figures 4B, C**) compared to PBS control, but only pOVA-PEG20 at a dose of 5 µg (based on peptide amount) was significantly more efficient than unconjugated pOVA.

#### Expansion of Pre-Existing Tregs or *De Novo* Induction?

**Figure 5:**
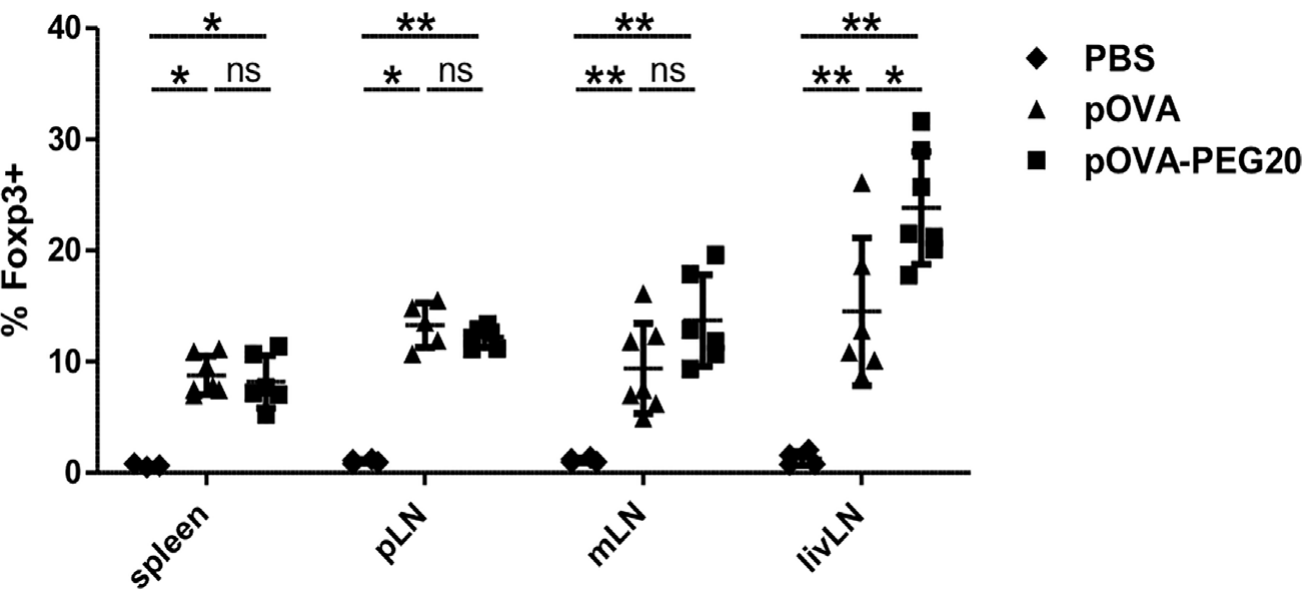
Administration of pOVA-PEG20 leads to *de novo* induction of Foxp3^+^ Tregs. Treg-depleted CD4^+^ T cells from DO11.10 mice were transferred into BALB/c mice 24 h prior to treatment with PBS (control), 5 µg of pOVA or equimolar amounts of pOVA-PEG20. Six days post peptide vaccination, spleen, pLN, mLN and livLN were isolated and Foxp3 expression was analyzed using flow cytometry. Data were pooled from two experiments and represent mean ± SD of % Foxp3^+^ cells gated on OVA-TCR^+^ CD4^+^ cells. n = 3–7. Statistical testing was performed using the nonparametric Mann Whitney test and Holm-Bonferroni correction for multiple comparisons. (ns) and all comparisons not marked were non-significant. *p < 0.05; **p < 0.01.

To investigate whether the increase of Treg frequency following pOVA-PEG20 treatment is due to *de novo* induction or to expansion of pre-existing Tregs, we transferred Treg-depleted OVA-specific CD4^+^ T cells prior to vaccination with pOVA or pOVA-PEG20. Six days after tolerogenic vaccination, secondary lymphoid organs [spleen, pLN, mLN, and liver-draining lymph node (livLN)] were isolated and Foxp3 expression of OVA-TCR^+^ CD4^+^ T cells was assessed using flow cytometry. pOVA and pOVA-PEG20 were able to induce Foxp3^+^ Tregs in all organs analyzed (**Figure 5**). The frequency of Foxp3^+^ Tregs reached similar levels following pOVA and pOVA-PEG20 administration in both spleen and pLN, whereas pOVA-PEG20 increased the Treg frequency in mLN (trend) and the livLN (significant) compared to pOVA. Interestingly, the frequency of Foxp3^+^ cells was not as high as observed by adoptive transfer of CD4^+^ populations including Tregs (**Figure 4**), suggesting that administration of PEGylated pOVA leads to both *de novo* induction of Foxp3^+^ Tregs and to expansion of pre-existing Tregs.

#### Reduction of Pro-Inflammatory Effector Cells

Another important mechanism of tolerance induction by systemic peptide encounter beside Treg induction is a diminished pro-inflammatory cytokine response, reflecting anergy of cells or depletion of the effector population.

**Figure 6:**
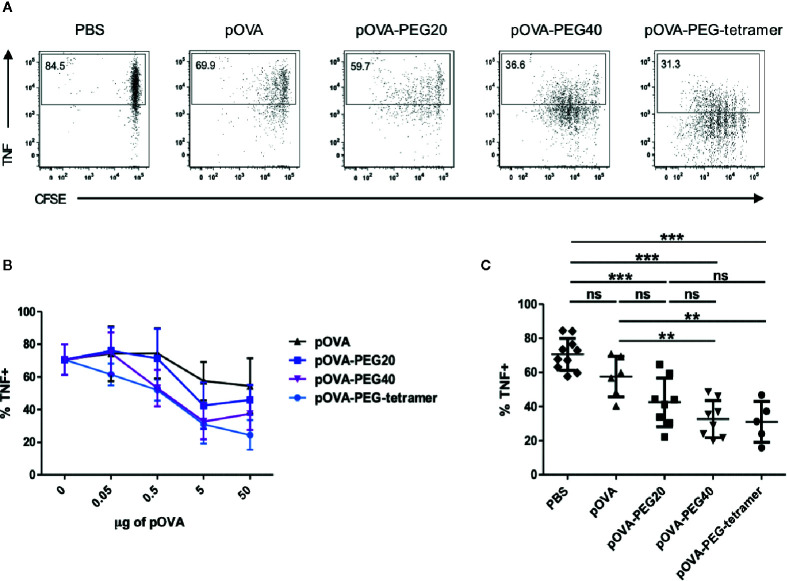
Reduced frequency of TNF^+^ among OVA-TCR^+^ T cells upon treatment with PEGylated pOVA. 24 h after transfer of OVA-specific CFSE-labeled CD4^+^ T cells, recipients were treated i.v. with PBS (control), pOVA or equimolar amounts of pOVA-PEG conjugates. After 6 days, TNF production was assessed by intracellular staining of restimulated cells. Splenocytes were gated on OVA-TCR^+^ CD4^+^ cells. **(A)** Representative FACS dot plots of TNF expression following tolerization with pOVA or pOVA-PEG conjugates at a dose of 5 µg (based on peptide amount). **(B)** Dose response curve. Mean ± SD of % TNF^+^ from at least two independent experiments. n = 4–6; PBS, n = 10. **(C)** Data for treatment with 5 µg of pOVA or equimolar amounts of PEGylated pOVA. Mean ± SD of % TNF^+^ cells from at least two independent experiments. Statistical testing was performed using the nonparametric Mann Whitney test and Holm-Bonferroni correction for multiple comparisons. ns, non-significant, **p < 0.01; ***p < 0.001.

At a dose of 5 µg, all PEGylated conjugates significantly reduced the frequency of TNF-producing cells among the OVA-specific T cells compared to PBS (**Figures 6A–C**). Interestingly, pOVA-PEG40 and pOVA-PEG-tetramer were superior in suppressing the TNF response already at lower doses. Under the conditions used (day 7), almost no IFN-γ or IL-10 -positive cells could be detected (**Supplementary Figure 3**).

**Figure 7:**
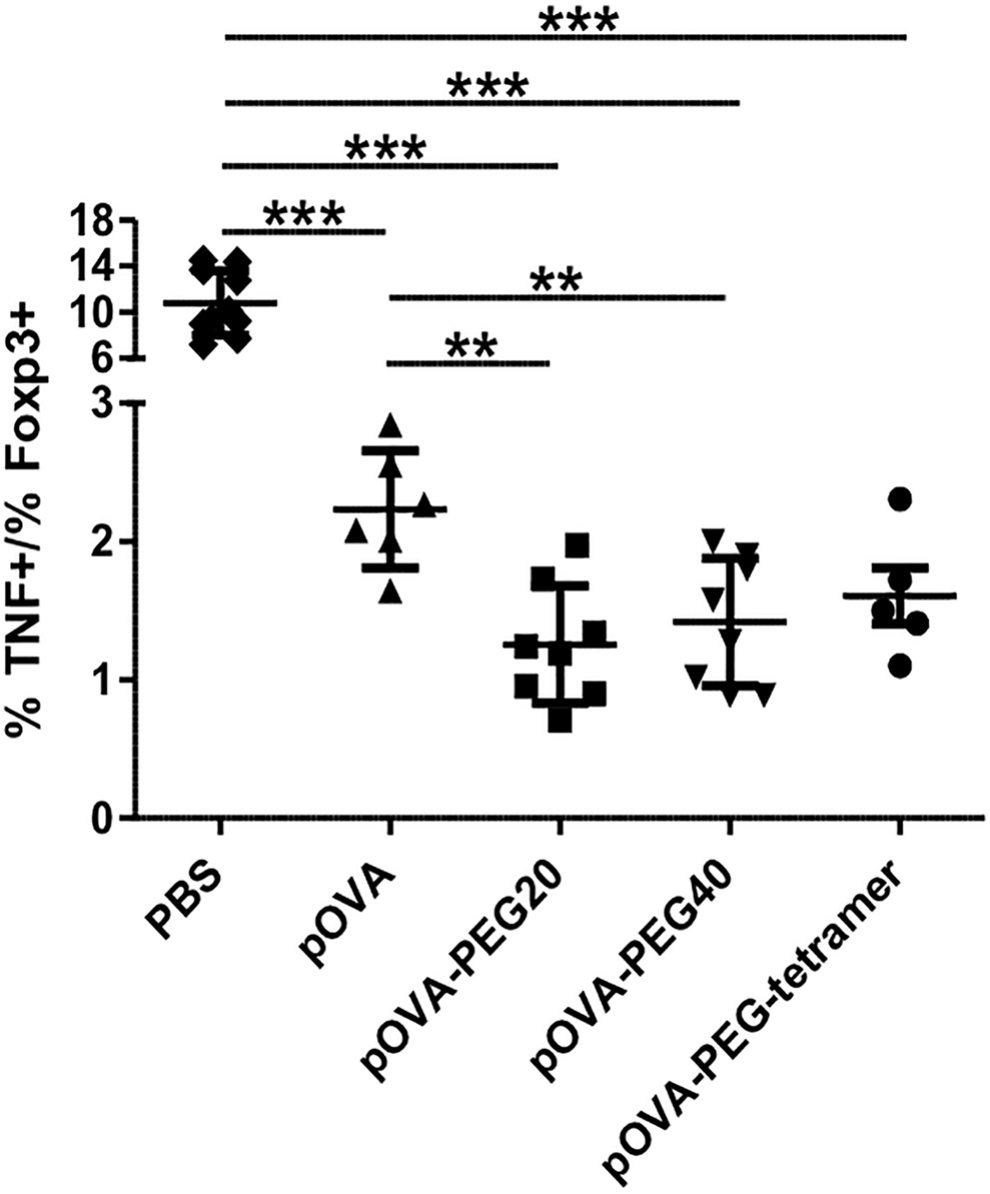
Reduced TNF/Foxp3 ratio upon treatment with pOVA-PEG conjugates. 24 h after transfer of OVA-TCR^+^ CFSE-labeled CD4^+^ T cells, recipients received PBS (control), 5 µg of pOVA or equimolar amounts of different pOVA-PEG conjugates. After 6 days, TNF production and Foxp3 expression were assessed using flow cytometry for spleen cells. The TNF/Foxp3 ratio gated on OVA-TCR^+^ CD4^+^ cells was calculated, reflecting the effector T cell/Treg ratio of responding cells. Mean ± SD of the ratio % TNF^+^/% Foxp3^+^cells from at least two independent experiments; n = 5–8; PBS, n = 10. Statistical testing was performed using the nonparametric Mann Whitney test and Holm-Bonferroni correction for multiple comparisons. **p < 0.01; ***p < 0.001.

The combined impact of tolerogenic vaccination on both induction/expansion of Foxp3^+^ Tregs and reduction of TNF-producing cells was visualized by the ratio of TNF-producing to Foxp3 expressing cells, representing the effector/regulatory T cell ratio *in vivo.* The lowest effector cell/Treg ratio was found in animals treated with pOVA-PEG20 at a dose of 5µg (pOVA-PEG20: mean ratio = 1.25; pOVA-PEG40: 1.42; pOVA-PEG-tetramer: 1.61; pOVA: 2.23; PBS: 10.79; **Figure 7**).

**Figure 8:**
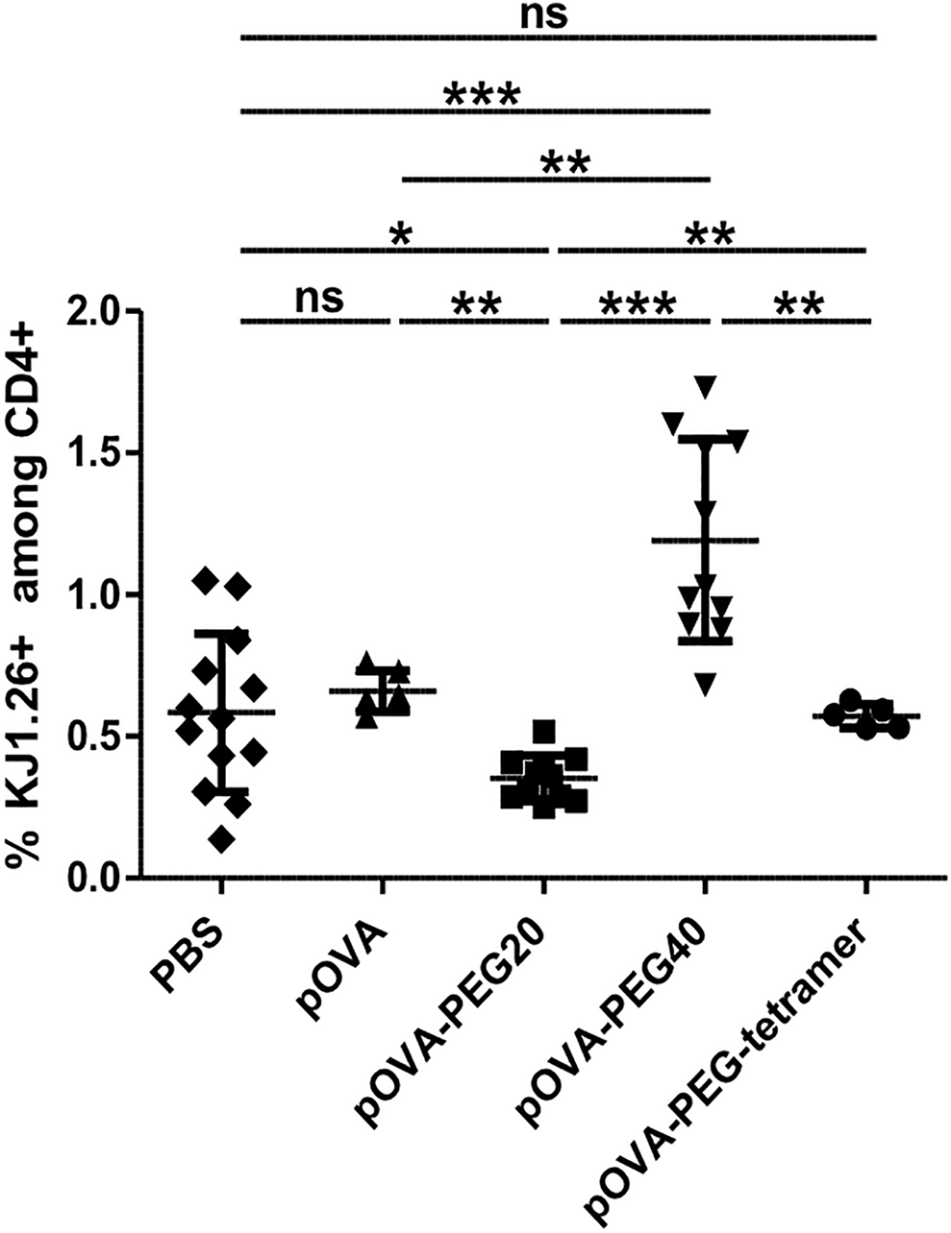
Administration of pOVA-PEG20 reduces the frequency of OVA-TCR^+^ CD4^+^ T cells among total CD4^+^ splenocytes. OVA-specific CD4^+^ cells were transferred into BALB/c mice. After 24 h, mice received i.v. PBS (control), 5 µg of pOVA or equimolar amounts of pOVA-PEG conjugates. After 6 days, the frequency of OVA-TCR^+^ CD4^+^ cells among total CD4^+^ T cells was assessed using flow cytometry. Mean ± SD of % KJ1.26^+^ cells (OVA-TCR^+^ T cells) among total CD4^+^ splenocytes from at least two independent experiments. n = 5–11; PBS, n = 13. Statistical testing was performed using the nonparametric Mann Whitney test and Holm-Bonferroni correction for multiple comparisons. ns, non-significant, *p < 0.05; **p < 0.01; ***p < 0.001.

Besides Treg expansion/induction and anergy, deletion of antigen-specific T effector cells by activation-induced cell death also dampens the immune response. While unconjugated pOVA was not reducing the frequency of OVA-specific cells among host cells (reflecting their absolute number) until day 6, pOVA-PEG20 significantly reduced their frequency compared to both PBS and non-modified pOVA (**Figure 8**), despite inducing a slightly stronger proliferation (**Figure 2**). Thus, vaccination with this conjugate is more efficient in triggering activation-induced death compared to native peptide or pOVA-PEG-tetramer vaccination where some death might outbalance induced proliferation. Further analysis revealed that deletion of OVA-TCR^+^ cells started to occur between day 3 and 4 post adoptive cell transfer (**Supplementary Figure 4**). In contrast to the other conjugates, administration of pOVA-PEG40 resulted in a net expansion of OVA-TCR^+^ CD4^+^ cells.

In summary, conjugation of the PEG20 turned out to be the most effective modification with regard to increasing Foxp3^+^ Treg frequencies, decreasing pro-inflammatory TNF producing cells and depletion of antigen-reactive cells. Therefore, pOVA-PEG20 was chosen as the most promising candidate for tolerogenic vaccination approaches.

#### Impact of pOVA-PEG20 on Pre-Existing Effector T Cells

A potential field of application of tolerogenic vaccination is the treatment of autoimmunity or allergy. In these cases, effector cells are already present and might not only be resistant to conversion into Tregs but also bear the risk of exacerbating the disease by activation of the effector cells, as observed in peptide vaccination studies in experimental autoimmune encephalomyelitis (EAE) or allergy models (10, 11).

**Figure 9:**
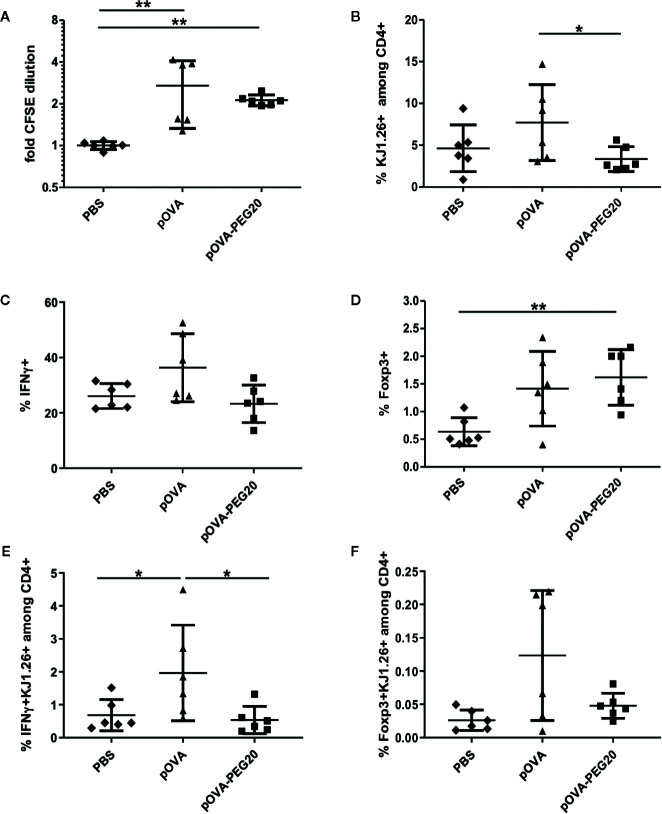
pOVA-PEG20 vaccination in presence of pre-existing T effector cells. *In vitro* generated OVA-specific Th1 cells were labeled with CFSE and transferred into BALB/c mice. After 24 h, recipients received i.v. PBS (control), 5 µg of pOVA or equimolar amounts of pOVA-PEG20. After four days, splenocytes were re-isolated and proliferation, IFN-γ and TNF production as well as Foxp3 expression was assessed using flow cytometry. **(A)** Proliferation (x-fold CFSE dilution). **(B)** OVA-TCR^+^ T cells among total CD4^+^ cells. **(C)** % IFN-γ producers among OVA-specific CD4^+^ cells. **(D)** % Foxp3^+^ cells among OVA-specific CD4^+^ cells. **(E, F)** Absolute number of antigen-specific IFNγ or Foxp3^+^ cells expressed as % of total CD4^+^. **(A–F)** Pooled data with mean ± SD from two independent experiments; n = 6. Non-marked comparisons are non-significant. Statistical testing was performed using the nonparametric Mann Whitney test and Holm-Bonferroni correction for multiple comparisons. (*) p < 0.05; (**) p < 0.01.

To analyze these questions, *in vitro* generated OVA-specific Th1 effector cells (**Supplementary Figure 5**) were transferred into BALB/c mice prior to peptide vaccination. Proliferation and expression of IFN-γ and Foxp3 were assessed on day 5. Although CFSE staining revealed a moderate proliferative response of the Th1 cells upon treatment of mice with pOVA-PEG20 or pOVA (**Figure 9A**), again proliferation appeared to be outbalanced by induced cell death after pOVA-PEG20 treatment as both total OVA-specific and the fraction of IFN-γ-producing cells was not increased upon pOVA-PEG20 treatment (**Figures 9B–E**).

The low frequency of Foxp3^+^ cells among transferred cells was moderately increased upon administration of pOVA-PEG20 (**Figures 9D, F**).

**Figure 10:**
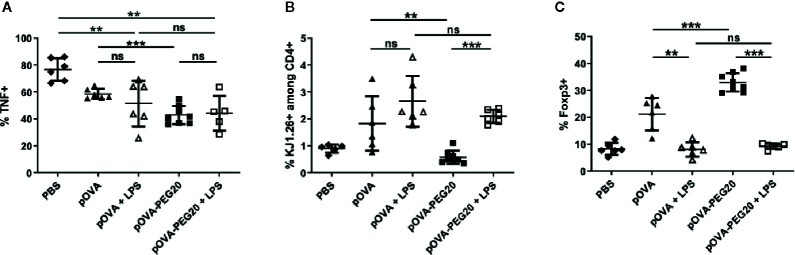
Administration of 50 µg of LPS does not enhance secretion of pro-inflammatory cytokines but inhibits induction/expansion of Foxp3^+^ Tregs upon treatment with pOVA-PEG20. 24 h after adoptive transfer of OVA-specific CD4^+^ T cells, recipients received i.v. PBS (control), 5 µg of pOVA or equimolar amounts of pOVA-PEG conjugates with or without additional LPS. After 6 days, splenocytes were isolated and Foxp3 expression as well as TNF production was assessed by flow cytometry. **(A)** % TNF^+^ cells gated on OVA-specific CD4^+^ cells, mean ± SD of data from three independent experiments. **(B)** % KJ1.26^+^ (OVA-specific) splenocytes among total CD4^+^ population; mean ± SD of pooled data from three independent experiments. **(C)** % Foxp3^+^ cells gated on OVA-specific CD4^+^ cells; mean ± SD of pooled data from three independent experiments. n = 5–6; pOVA-PEG20, n = 8. Statistical testing was performed using the nonparametric Mann Whitney test and Holm-Bonferroni correction for multiple comparisons. ns, non-significant, **p < 0.01; ***p < 0.001.

To investigate the impact of inflammatory conditions, we analyzed the effect of lipopolysaccharide (LPS) application on the response of transferred unmanipulated OVA-TCR^+^ CD4^+^ cells. While LPS did not further increase the frequency of TNF-producers, the expansion of OVA-TCR^+^ T cells upon vaccination with pOVA-PEG20 was stronger in presence of LPS (**Figures 10A, B**). Remarkably, the increase in Foxp3^+^ cells upon vaccination with pOVA or pOVA-PEG20 was completely suppressed in presence of LPS (**Figure 10C**).

#### Tolerogenic Effects in a DO11.10 Transfer-Based DTH Model

**Figure 11:**
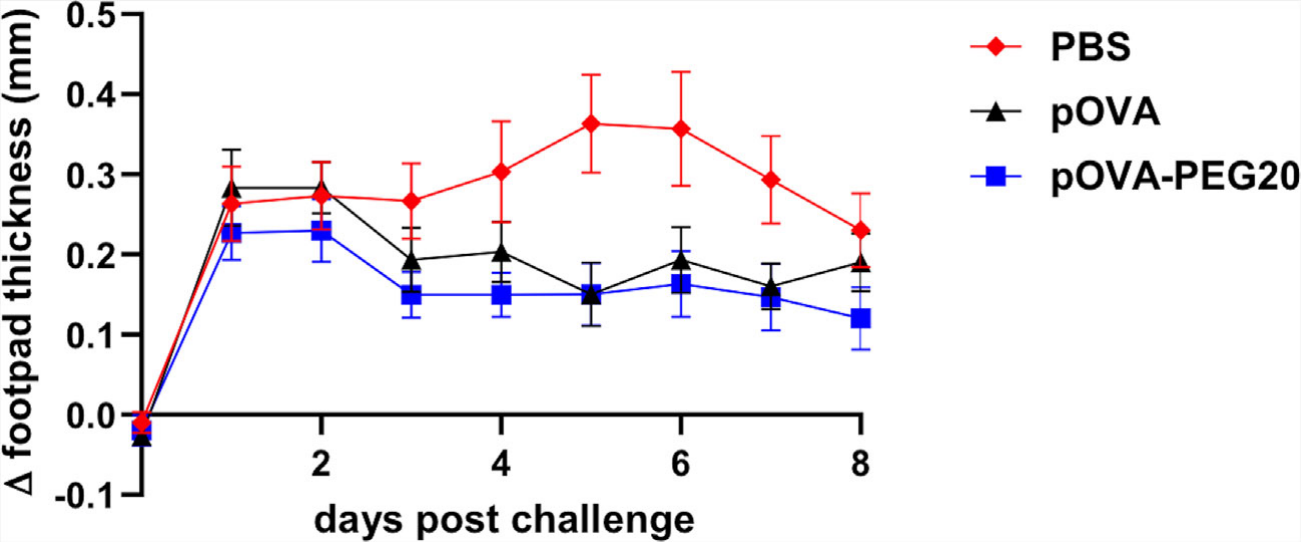
Vaccination by either pOVA or pOVA-PEG20 confers partial tolerance in a Th1-driven DTH model. OVA-specific CD4^+^ cells were adoptively transferred into BALB/c mice, which were treated i.v. with PBS (control), 5 μg of pOVA or equimolar amounts of pOVA-PEG20 24 h later. On day 7, *in vitro* generated OVA-specific Th1 cells were transferred into the recipients. After 24 h, pOVA in IFA or the control, PBS/IFA, were injected into the left or right hind footpad, respectively. The difference in footpad thickness after challenge was monitored for eight consecutive days. Statistical testing was performed using the nonparametric Mann Whitney test and Holm-Bonferroni correction for multiple comparisons. Difference between pOVA-PEG20 and pOVA was significant with adjusted p = 0.03. Data represent mean of Δ footpad thickness ± SEM (n = 15).

To investigate whether pOVA-PEG20 is able to prevent the development of an inflammatory reaction *in vivo*, we used a DTH model based on the transfer of pre-formed Th1 cells, intra-footpad challenge with pOVA and measurement of footpad swelling for 8 days. As shown in **Figure 11**, only a slightly more efficient suppression by pOVA-PEG 20 compared to pOVA is observed (Mann Whitney U test, adjusted p = 0.03) in this setting. As discussed below, a clearer superiority of PEGylated peptide over native peptide in tolerance induction was found in a more physiological EAE-model studied in parallel (35).

### Antigen Uptake and Presentation by Antigen-Presenting Cells

The type and status of APC that present antigen in a given context can play a role in the induction of tolerance (36, 37). To investigate which APC population is able to take up pOVA-PEG20, pOVA-PEG20 was conjugated to the fluorochrome fluorescein isothiocyanate (FITC) and the FITC^+^ fractions of major subgroups of APC were identified by flow cytometry. Uptake of pOVA-PEG-FITC was observed by CD11c^+^ cells (DC) and CD11b^+^CD11c^-^ cells (macrophages) and, to a lesser extent, by B cells in all analyzed secondary lymphoid organs (**Supplementary Figures 7, 8**), in partial agreement with previous studies of antigen presentation upon oral administration (38).

Besides APCs from hematopoietic origin, also non-hematopoietic cells are able to present antigens to T cells *via* the major histocompatibility complex class II (MHCII) (34, 39–41). Especially liver sinusoidal endothelial cells have been associated with tolerogenic responses (34, 42). To investigate their role in presentation of pOVA-PEG20, chimeric mice devoid of hematopoietic MHCII^+^ APC were used. Chimeric mice were generated by irradiation of wt C57BL/6 mice and reconstitution of the hematopoietic compartment with BM cells from MHCII^-^/^-^ mice. After successful reconstitution, CFSE-labeled OVA-specific MHCII^+^ depleted CD4^+^ cells isolated from transgenic OTIIxB6.PL mice were transferred into the BM chimeras 24 hours prior to pOVA-PEG20 administration. After 6 days, OVA-specific CD4^+^ splenocytes were isolated, and proliferation and Foxp3 expression were assessed by flow cytometry.

**Figure 12:**
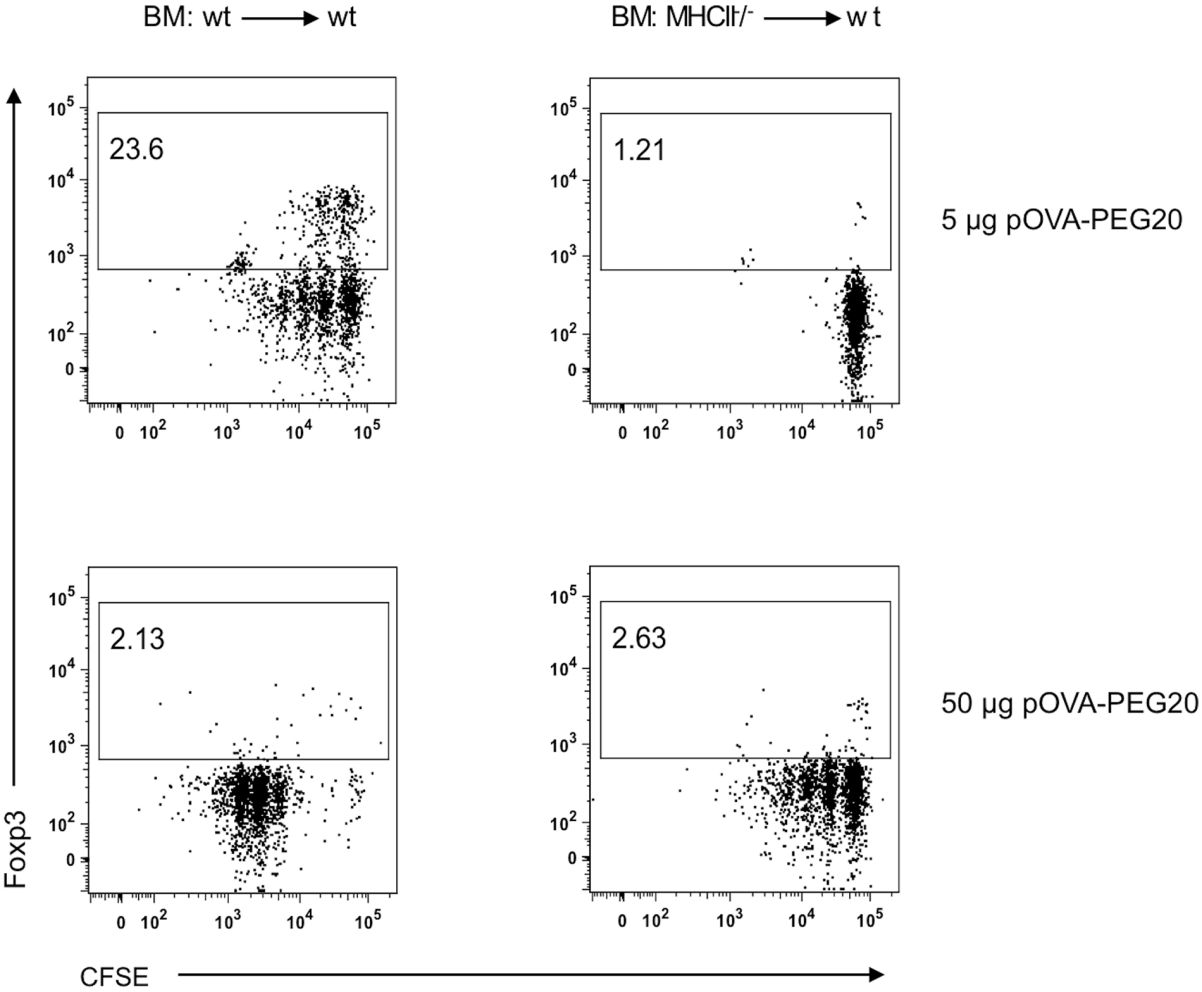
Non-hematopoietic APCs present pOVA-PEG20 and induce activation of OVA-specific T cells at high doses. OVA-specific CD4^+^ MHCII^-^/^-^ cells, isolated from OTIIxB6.PL (CD90.1^+^) mice, were labeled with CFSE and transferred into controls or MHCII deficient BM (bone marrow) chimeras. After 24 h, recipients received 5 or 50 µg (based on peptide amount) of pOVA-PEG20. Proliferation as well as Foxp3 expression was assessed using flow cytometry on day 7. Splenocytes were gated on donor (CD90.1^+^) OVA-specific CD4^+^ T cells. Representative FACS dot plots from at least two independent experiments. wt: wild type.

5 µg of pOVA-PEG20 (based on peptide amount) were sufficient to induce proliferation of OVA-TCR^+^ T cells in MHCII^+^/^+^ (wt) control BM chimeras, whereas the equal dose did not activate OVA-specific cells in MHCII^-^/^-^ BM chimeras (**Figure 12**). Here, a tenfold dose (50 µg) was required to induce T cell activation. In contrast to wt BM chimeras, Foxp3 Treg expansion was not observed in MHCII^-^/^-^ BM chimeras, neither at 5 nor at 50 µg, indicating that non-hematopoietic APCs are able to present pOVA-PEG20 and thereby activate OVA-specific T cells, but do not mediate induction or expansion of Foxp3^+^ Tregs.

## Discussion

Induction of tolerance by antigenic peptides has been notoriously inefficient and, in some cases, even aggravating disease. We here studied whether conjugation of antigenic peptides to a polyethylene glycol moiety can improve their tolerogenic potential by prolonging half-life and by reducing effector cell stimulation.

Coupling of peptide to PEG might prevent their presentation by MHC. DO11.10 T cells recognize a core epitope (329–339) with the primary and secondary TCR contact residues 333 and 331, respectively (43). N-terminal PEGylation of the OVA-peptide CG-323–339 poses the bulky PEG chain 8–10 amino acid residues away from the recognition motif. Nevertheless, we observed a strongly reduced stimulatory capacity of pOVA-PEG conjugates compared to the native peptide in the *in vitro* assay. As to be expected, the tetramer conjugate was the most active compound *in vitro*, while, surprisingly, also the PEG40 was significantly more active than the PEG20 conjugate.

*In vitro*, peptides are predominantly directly loaded onto MHCII; this process could be impaired by the attached PEG moiety. Whether presentation of the conjugates requires intracellular or extracellular cleavage of the PEG-entity from the core peptide remains to be analyzed. Increasing the PEG size from 20 to 40 kDa did not further reduce the biologic activity *in vitro*.

In contrast to the reduced *in vitro* stimulatory capacity of pOVA-PEG conjugates, a stronger proliferative response of T cells was elicited *in vivo.* The magnitude of this effect was dependent on structure and size of the PEG molecule. Thus, impairment of presentation is compensated for by mechanisms increasing the biologic efficacy *in vivo*.

The stimulatory capacity of native peptides *in vivo* is restricted by enzymatic degradation and rapid renal clearance in the organism. This can be counteracted by PEGylation (44). We therefore hypothesized that PEGylation of peptide would lead to an extended bioavailability *in vivo*. To prove this hypothesis, the transgenic DO11.10 adoptive transfer model was modified in that conjugated or native peptide was given three days before transfer of reactive T cells, so that the retention time of the peptide within the body would play a critical role. Under these conditions, only marginal T cell activation was observed upon vaccination with non-modified pOVA and pOVA conjugated to a small linear 12 kDa PEG molecule, suggesting a complete systemic clearance within three days. In contrast, conjugation of pOVA with PEG20, PEG40, or as a tetramer significantly prolonged the bioavailability of the antigen. Modification of biomolecules with linear PEG molecules increases their hydrodynamic volume and thereby affects glomerular filtration (27). In agreement with our observations, Jorgenson and Moller previously showed that a minimum of 20 kDa is needed for a significant retardation of renal clearance (45).

Interestingly, administration of equimolar amounts (related to peptide) of pOVA-PEG-tetramer induced the strongest proliferation compared to other peptide-PEG-conjugates in spleen, although the molecular weight of the pOVA-PEG-tetramer is in total approximately 11 kDa, i.e., below the minimum of 20 kDa. According to Veronese and colleagues, conjugation of branched PEGs achieves better proteolytic resistance than PEGylation with linear PEG of the similar net molecular weight (46). Alternatively, the tetravalent nature of pOVA-PEG tetramer could lead to an improved avidity in the MHC-peptide-TCR complex.

It should be noted that reported values for the serum half-life of PEGylated peptides, even of higher molecular weight, are rather in the range of <1 day (47). In the present study, PEGylation (except with PEG12) of the OVA-peptide allowed recognition by T cells beyond 72 h. Beside the option that declined concentrations might still be sufficient for activation, improved uptake, tissue sequestration or intracellular storage of the conjugates can contribute to the extended bioavailability *in vivo*. Indeed, when FITC-conjugated pOVA-PEG20 was applied, DCs, macrophages, and, to a lesser extent, B cells became labeled, indicating uptake by these APC.

The key goal of inverse vaccination is the modulation of the immune system towards antigen-specific tolerance. We therefore investigated in this study whether mechanisms involved in tolerance such as Treg expansion/induction, anergy or depletion of antigen-specific cells are targeted by vaccination with PEGylated peptide.

Tregs can be peripherally induced by various protocols including oral (48, 49), intranasal (50), or intravenous (51) antigen administration. We here demonstrate that the systemic administration of PEG20-conjugated pOVA at a dose of 5 µg (based on peptide amount) results in a significantly elevated frequency of pOVA-TCR^+^ Foxp3^+^ Tregs compared to unconjugated pOVA. While pOVA-PEG40 and pOVA-PEG-tetramer most effectively induced proliferation, Treg frequencies did not exceed those upon pOVA exposure. This supports the notion that size and structure of the PEG-scaffolds affects both quality and quantity of the immune response. This was also found in our previous study using a different synthetic scaffold, dendritic oligoglycerol (OG)- and polyglycerol (PG)-nanoparticles loaded with OVA-peptide. These conjugates also induced Foxp3^+^ Tregs (20), but chemistry, size, and structure of the synthetic carrier played a critical role for their tolerogenic potential, same as found by (18, 19, 22).

In accordance to previous studies using native peptide (51), high doses of antigen were less effective in inducing Tregs, while in our study doses below 5 µg (not studied by these authors) were also less effective, resulting in a bell-shape dose-response curve. The superior effect of PEG20-conjugates was highly significant only at this dose, highlighting the importance of optimal dosing in tolerance induction.

In order to analyze whether the increased frequencies of Foxp3^+^ Tregs are due to *de novo* induction or result from expansion of pre-existing Tregs, CD25^+^ Tregs were depleted prior to adoptive transfer of OVA-specific CD4^+^ cells. Significant numbers of Foxp3^+^ Tregs were found upon vaccination with pOVA or pOVA-PEG20 in all organs analyzed, indicating *de novo* generation from naïve cells. We can exclude that this is due to small numbers of Tregs remaining in the Treg-depleted preparation, as a previous study on oral tolerization (49) demonstrated that the small percentage of Tregs remaining in Treg-depleted cells is not preferentially expanding. As frequencies of Foxp3^+^ cells induced were lower than for total CD4^+^ cells, it can be concluded that both *de novo* induction and expansion of pre-existing Tregs might account for the total frequency of up to 30% Tregs in spleen upon vaccination with pOVA-PEG20.

Interestingly, in the present study *de novo* induction of Foxp3^+^ Tregs was most prominent in the liver-draining lymph node (livLN). Several studies have shown a pivotal role for the livLN in generation of suppressive CD25^+^ Tregs following oral antigen administration (49, 52) and provided evidence that the microenvironment of the liver is biased towards tolerogenic responses (42, 53, 54). In particular, the liver sinusoidal endothelial cells (LSEC) have been shown to down-regulate CD8^+^ T cell responses (34, 53). Controversial findings are reported regarding the capacity of LSEC to induce Foxp3^+^ Tregs: Carambia and colleagues observed a TGF_β_-dependent induction of CD4^+^ Foxp3^+^ Tregs by LSECs and demonstrated that antigen-loaded nanoparticles targeting LSECs could efficiently prevent the onset or ameliorate established EAE, mediated by Treg induction (55, 56). In contrast, Kruse et al. observed that activation of CD4^+^ cells using *ex vivo* LSECs as APC or a MHCII^-/-^ BM chimeric mouse model resulted in anergic and suppressive T cells which were Foxp3^-^ but able to suppress inflammation in autoimmune hepatitis (34).

We therefore studied here whether non-hematopoietic cells were involved in the activation of CD4^+^ T cells and conversion into Tregs following administration of pOVA-PEG20. In BM-chimeric mice lacking MHCII^+^ hematopoietic APC, the standard dose of 5 µg of peptide did not lead to a significant T cell response, while the tenfold dose induced proliferation. In accordance with (34), the T cells proliferating at high peptide dosis in the chimeric mice largely lacked Foxp3. We conclude that the non-hematopoietic cells including LSEC do not significantly contribute to the (Foxp3^+^) Treg-inducing capacity of pOVA-PEG conjugates.

Experiments analyzing the uptake of fluorescently labeled pOVA-PEG did not provide hints for a selective uptake by a certain subset of conventional APC. However, this does not exclude that a specific type of APC is crucial for the conversion of naïve T cells into Tregs.

Various studies have demonstrated that induction of Tregs as well as their function becomes impaired in the presence of inflammatory signals. (51, 57, 58). In agreement with these findings, pOVA-PEG20-mediated induction/expansion of Foxp3+ Tregs was suppressed in presence of LPS. Furthermore, as previously reported (59), Th1 cells were largely resistant to conversion into Tregs, despite a minute increase in Foxp3^+^ cells. These features of tolerance induction let predict that therapeutic application of pegylated peptides will be feasible in a preventive setting, but for established disease, at most during remission phases or under combinatorial treatment strategies combining suppression of inflammation with induction of Tregs.

Beside dominant suppression mediated by Tregs, peripheral tolerance (3) also involves deletion of antigen-specific cells by activation-induced cell death (60) and anergy (4), e.g., failure of antigen-reactive cells to produce pro-inflammatory cytokines. The data of this study point to a partial deletion of antigen-specific effector cells upon injection of pOVA-PEG, as also found upon vaccination with multimerized T cell epitopes (61) and T cell epitopes linked to parasite-derived tandem repeats (17).

Inflammatory cytokines such as TNF are major players in pathogenicity and TNF is a major target for treatment, e.g., in rheumatoid arthritis (62). Loss of effector functions such as secretion of cytokines has proposed to play a significant role in peripheral tolerance (4, 59). We studied the impact of peptide vaccination on TNF production as a flagship of inflammatory cytokines. We found that administration of pOVA-PEG conjugates resulted in reduced frequencies of TNF-producing cells after re-stimulation with PMA/ionomycin. pOVA-PEG40 and pOVA-PEG-tetramer yielded the greatest reduction of TNF-producing cells, correlating with an increased stimulatory capacity. To assess the combined impact on both induction/expansion of Tregs and reduction of TNF-producing cells, we calculated the Foxp3/TNF ratio upon vaccination. pOVA-PEG20 most efficiently shifted this ratio in favor to regulatory immunomodulation compared to native pOVA and other pOVA-PEG conjugates. A similar diminished TNF- and increased Treg response was found upon vaccination with peptide conjugated to a parasite-derived carrier module (17). It remains to be shown whether these inhibitory effects on effector/inflammatory pathways are robust enough to achieve a successful therapy in ongoing autoimmune disease.

For application of tolerogenic vaccination strategies, safety is of outstanding importance. In established disease, antigen-specific effector/memory cells, such as polarized Th1 cells, might be present, even when not activated. Previous studies reported adverse effects after administration of soluble autoantigen peptides due to activation or reactivation of effector/memory cells (10, 11). It was therefore important to analyze whether PEG-conjugated peptides are advantageous over free peptides in this respect. In fact, pOVA-PEG20 did not lead to an exacerbation of the immune response in mice adoptively transferred with Th1 cells to mimic an established disease. In spite of significant proliferation induced by both pOVA and pOVA-PEG20, only the latter did not give rise to increased frequencies and numbers of IFNγ-producing Th1 cells. It appears that the PEGylated peptide is enforcing mechanisms leading to activation-induced cell death rather than productive activation of effector cells. The reasons for this favorable property have yet to be elucidated.

In conclusion, the present investigations in the OVA-model suggest that vaccination with PEG-conjugated peptides is significantly more effective in inducing antigen-specific Tregs and less prone to adverse activation effects on the effector side than vaccination with free peptide. In addition, vaccination with the conjugates was favorable with respect to shifting the ratio between effector cells and regulatory cells. Thus, conjugation of autoantigen- or allergen-derived immunodominant peptides to PEG might be promising to increase efficacy and safety of tolerogenic vaccines.

While in a DTH-model based on Th1 cell transfer before challenge we observed only a minor improvement of the tolerogenic potential already shown by the native peptide upon use of the PEGylated conjugate, we found, in a forthcoming study, that vaccination with PEGylated myelin-derived peptide MOG is much more effective in preventing disease in a mouse EAE-model for human multiple sclerosis (35). Thus, the value of the concept has finally to be proven in relevant preclinical models.

Compared to nanoparticle-based scaffolds, PEGylation appears to be advantageous as production and quality control might be easier. However, it has to be mentioned that the existence of antibodies in humans against PEG (mostly resulting from widespread use of PEG in cosmetics etc.) raised some concern (63). While such antibodies appeared not to interfere e.g., in trials using PEGylated interferon, in other cases biokinetics of PEGylated bacterial enzymes were found to be negatively affected (63, 64). Whether this issue will be of relevance for the type of conjugates used in our study remains to be analyzed.

We here confirmed repeated findings that a tolerogenic response is inhibited under inflammatory conditions as simultaneous delivery of LPS suppressed Treg generation. Thus, the capacity of a given vaccine not only to induce an antigen-specific Treg induction/expansion, but to suppress simultaneously and efficiently the activation of existing memory/effector cells or to suppress non-specific inflammatory signals might be crucial.

To achieve a maximum of both Treg-inducing and anti-effector and anti-inflammatory effects, different strategies can be considered:

- Combination of Treg-inducing agents such as peptide-PEG conjugates with agents or treatments suppressing effector mechanisms. The anti CD3 therapy already being in clinical testing is such a candidate, as it appears to cause the deletion of effector/memory cells (65).
- Combining the tolerogenic peptide vaccination with anti-inflammatory or Treg-promoting agents by their encapsulation in antigen-loaded nanoparticles. For this approach promising results have recently been reported (21, 23, 66).
- Certain types of nanoparticles appear by itself to induce down-regulation of inflammatory signaling pathways in accessory cells; this might explain their encouraging preclinical efficacy in models of autoimmune disease (19, 22, 67).

In conclusion, the old dream of inducing tolerance as an exclusively specific therapy for autoimmune diseases might come a step nearer by these recent developments.

## Data Availability Statement

All datasets generated for this study are included in the article/**Supplementary Material**.

## Ethics Statement

The animal study was reviewed and approved by Landesamt für Gesundheit und Soziales (LAGeSo), Turmstr. 21, 10559 Berlin.

## Author Contributions

JP, MS, UH, RK, FL, and AH conceived and designed the experiments. BT and RK synthesized and analyzed reagents. JP, MS, and UL performed the experiments. JP, MS, and PD analyzed the data. JP and AH wrote and edited the manuscript. All authors contributed to the article and approved the submitted version.

## Funding

This work was supported by the German Research Foundation (CRC 650 TP1), by the Federal Ministry of Education and Research (Innovative Therapies -01GU0722-) and the Federal Ministry for Economic Affairs and Energy (ZIM -KF 2441003SK1-).

## Conflict of Interest

The authors BT, RK, and FL were employed by Celares GmbH, a company offering contract development services to the pharmaceutical industry.

The remaining authors declare that the research was conducted in the absence of any commercial or financial relationships that could be construed as a potential conflict of interest.
