# Supplementary material for: Tolerogenic Immunomodulation by PEGylated Antigenic Peptides
Source: Front Immunol. 2020 Oct 9;11:529035. doi: 10.3389/fimmu.2020.529035 (PMC7581722; doi:10.3389/fimmu.2020.529035)
Supplement: Supplementary file 1 [file DataSheet_1.pdf]

Supplementary Material

pOVA-PEG (linear)

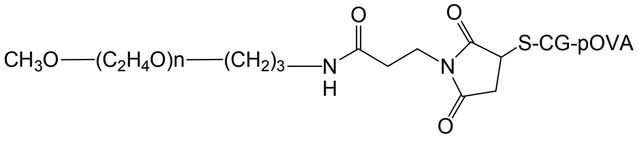

n= approx. 450 or 900

pOVA-PEG (tetramer)

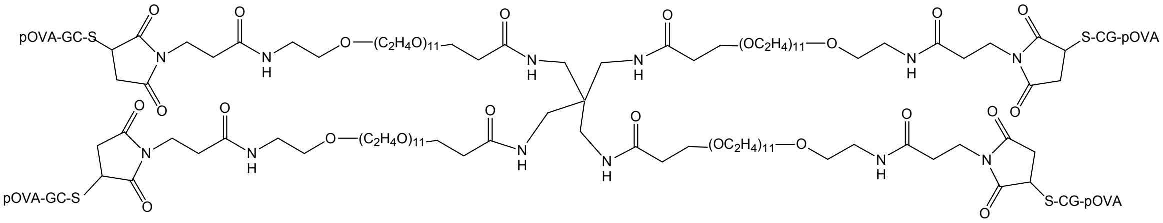

pMOG-PEG

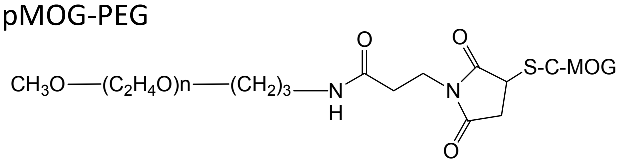

n= approx. 450

Supplementary Figure 1:

Formulas of PEG-peptide conjugates used.

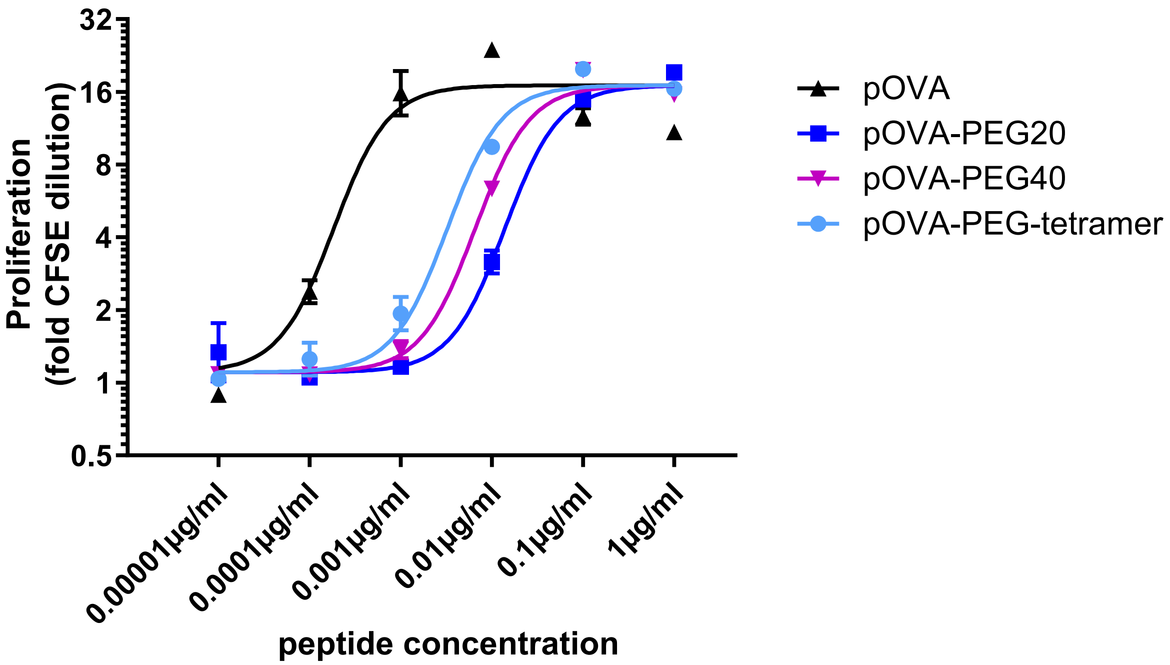

Supplementary Figure 2

Stimulatory capacity of PEGylated peptides *in vitro:* Fitted dose-response curves of peptide and conjugates.
Higher concentrations of PEGylated peptides were required to induce half-maximal proliferation compared to unmodified pOVA with 21 to 91-fold higher effective concentration (EC50).
EC50 pOVA-PEG20: 91.0 (fold EC50 of pOVA); 95% confidence interval (CI): 26.0-156.0;
EC50 pOVA-PEG40: 43.9; CI: 11.4-76.4;
EC50 pOVA-PEG-tetramer: 21.0; CI: 5.4-36.7).
Dose-response analysis was performed using GraphPad Prism version 7. EC50 shift was estimated by global nonlinear fitting of the default Hill model, assuming same minimal and maximal proliferation and variable Hill-slope for each conjugate. The number of cell divisions, that is log2 of the dilution factor of CFSE, was used as the proliferation output. Statistical differences between the EC50 values of the conjugates were computed applying the sum-of-squares F-Test for the unconstrained model and EC50 shift of 1. All dose-response curves were significantly different from each other with $p < 0.001$.

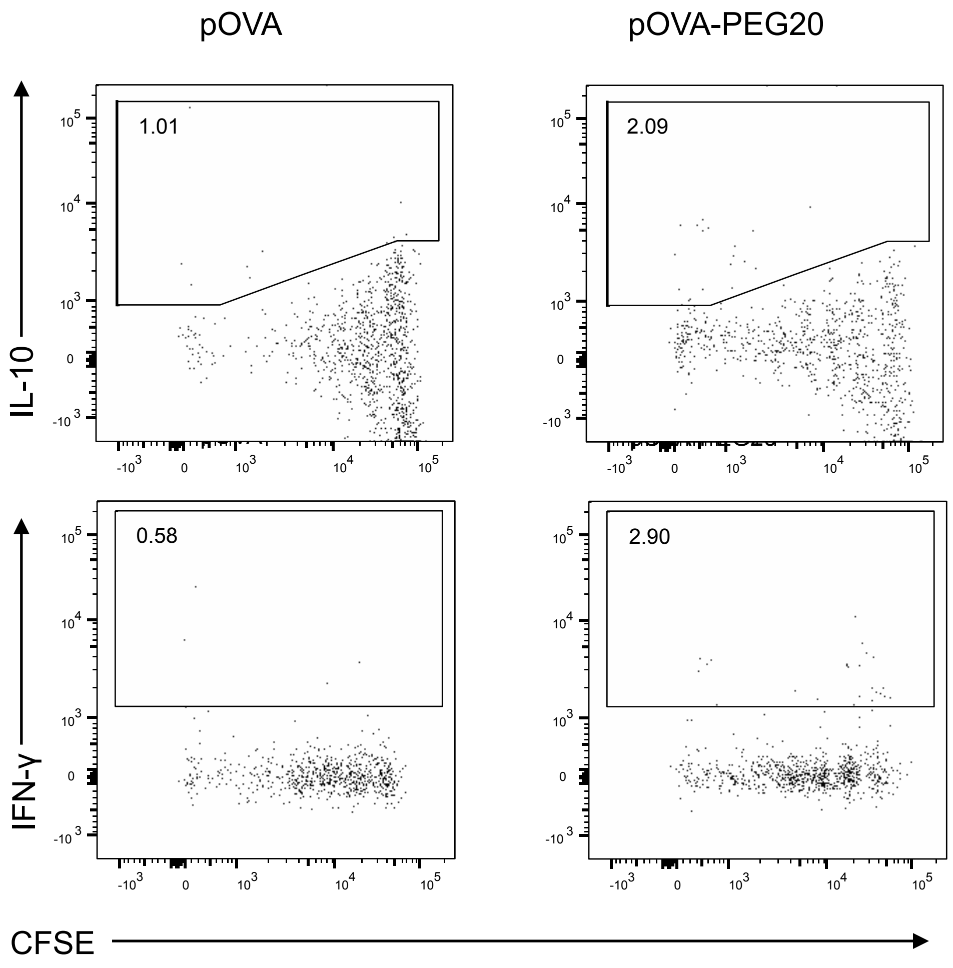

Supplementary Figure 3

Representative IL10 and IFNγ staining (after restimulation) upon stimulation with 5 µg pOVA and pOVA-PEG20 *in vivo*; day 7.

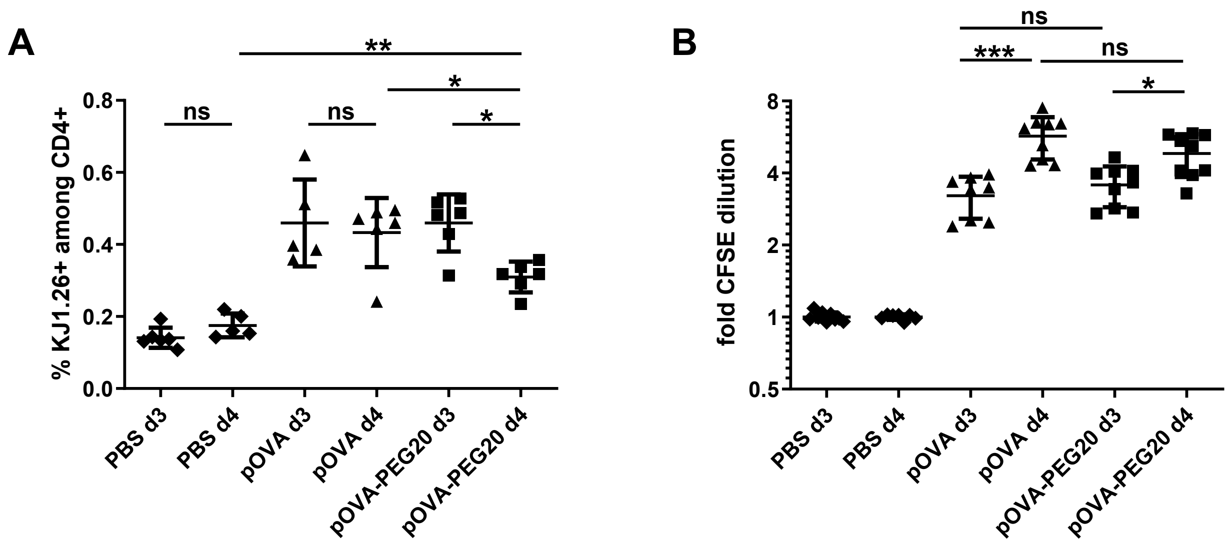

Supplementary Figure 4:

Faster decline in the number of OVA-specific CD4$^+$ T cells following pOVA-PEG20 after day 3, despite ongoing proliferation.

24 h after adoptive transfer of OVA-specific CFSE-labeled CD4$^+$ T cells, recipients received *i.v.* PBS (control), 5 µg pOVA or equimolar amounts of pOVA-PEG20. Splenocytes were isolated on day 3 (d3) and day 4 (d4) and analyzed using flow cytometry. (A) % KJ1.26$^+$ cells (OVA-specific T cells) among total CD4$^+$ cells. Mean ± SD of (n = 6). One representative of two independent experiments is shown. (B) Proliferation (mean x-fold CFSE dilution ± SD) of OVA-specific CD4$^+$ T cells. Data from two independent experiments, n = 7-9. Statistical testing was performed using the nonparametric Mann Whitney test and Holm-Bonferroni correction for multiple comparisons.

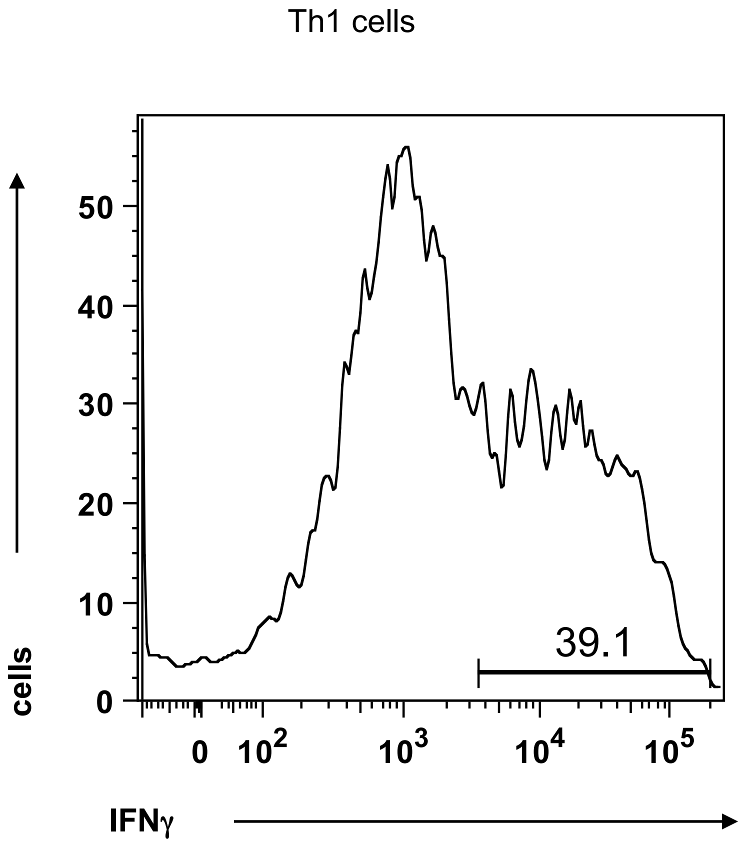

Supplementary Figure 5:

Impact of pOVA-PEG20 vaccination in presence of pre-existing T effector cells.
Representative histogram of IFN-γ expression of *in vitro* generated OVA-specific Th1 cells on day 5 of culture before adoptive transfer.

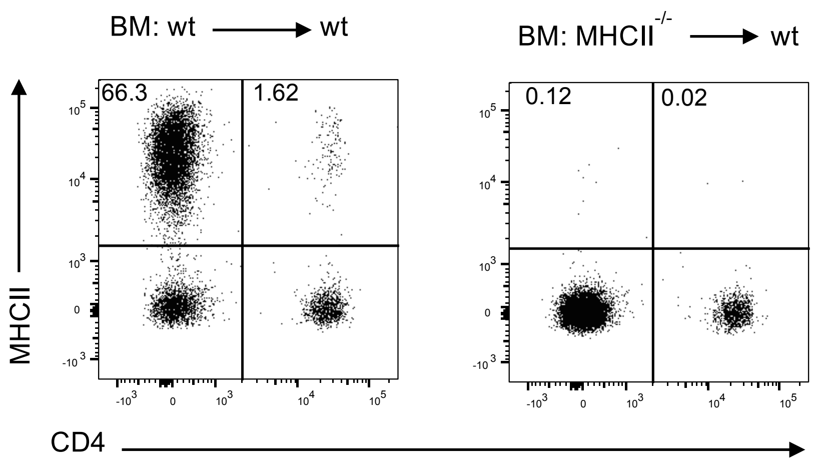

Supplementary Figure 6:

Control staining of blood leucocytes for MHCII, 8 weeks after reconstitution of BM-depleted B6 mice with BM from MHCII$^{-/-}$ mice.

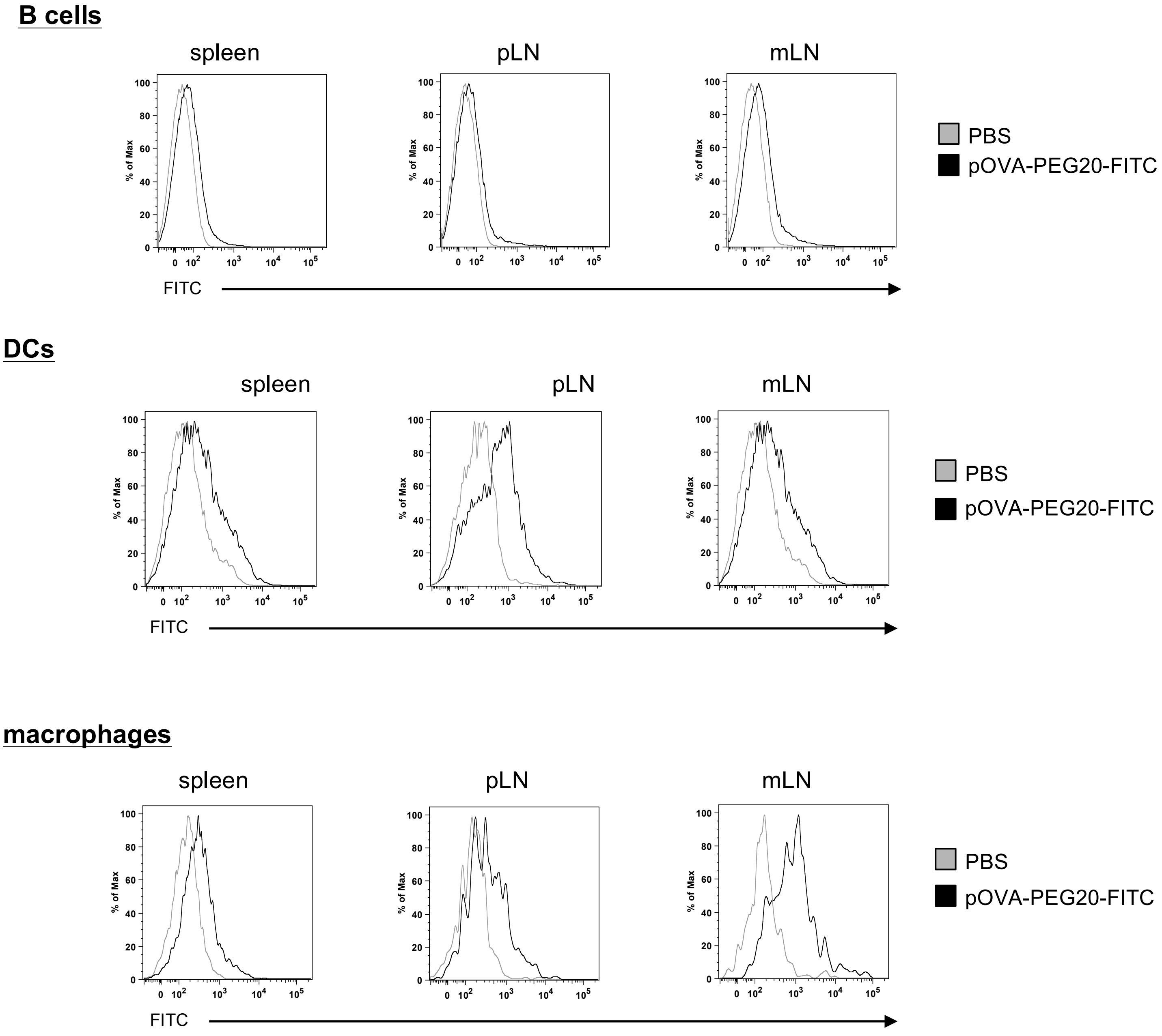

Supplementary Figure 7:

Uptake of pOVA-PEG20 by different hematopoietic APC subsets.
Mice received PBS (grey) or 100 µg (based on peptide amount) pOVA-PEG20 (black) conjugated to FITC *i.v.*. After one hour mice were sacrificed and secondary lymphoid organs were isolated. Cells were stained with surface markers and analyzed flow cytometry. APC populations were characterized as follows: B cells ($CD19^{+}$), DCs ($CD11c^{+}$) and macrophages ($CD11c^{-}$ $CD11b^{+}$). Representative histograms of FITC-intensity. Representative data from two independent experiments.

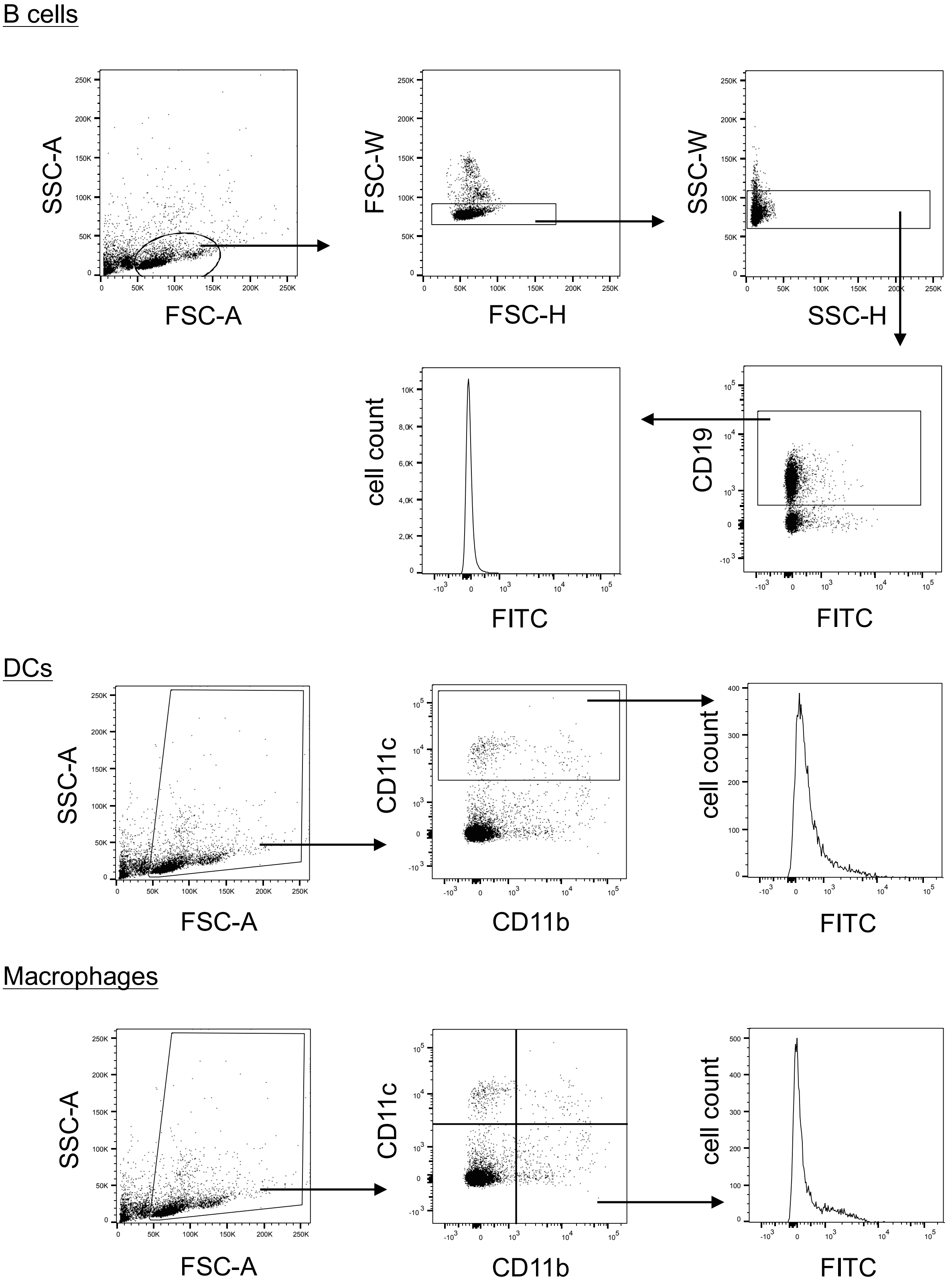

Supplementary Figure 8
Gating strategy for the identification of cells taking up pOVA-PEG20-FITC
